# Supplementary material for: Teacher support for generative AI use and design learning outcomes in fashion design education: A structural equation modeling study
Source: PLoS One. 2026 May 6;21(5):e0347428. doi: 10.1371/journal.pone.0347428 (PMC13148677; doi:10.1371/journal.pone.0347428)
Supplement: Attachment — Submitted filename: annotated PONE-D-25-50100.pdf [file pone.0347428.s002.pdf]

PLOS ONE

Teacher Support for Generative AI Use and Design Learning Outcomes in Fashion Design Education: A Structural Equation Modeling Study

--Manuscript Draft--

**Manuscript Number:**	PONE-D-25-50100
**Article Type:**	Research Article
**Full Title:**	Teacher Support for Generative AI Use and Design Learning Outcomes in Fashion Design Education: A Structural Equation Modeling Study
**Short Title:**	Generative AI Use and Design Learning Outcomes in Fashion Design Education
**Corresponding Author:**	zhiyi zhang, Ph.D.<br>University of Leeds<br>LEEDS, West Yorkshire UNITED KINGDOM OF GREAT BRITAIN AND NORTHERN IRELAND
**Keywords:**	Generative artificial intelligence; teacher support; design learning outcomes; fashion design education; creative design capability
**Abstract:**	The rapid integration of generative artificial intelligence (AI) in creative education necessitates empirical investigation of support mechanisms facilitating effective technology adoption and learning outcomes. This study examines the structural relationships among teacher support dimensions, generative AI usage behavior, creative design capability, and design learning outcomes in fashion design education contexts. Drawing on Technology Acceptance Model and Social Cognitive Theory, we developed and tested a chain mediation model using structural equation modeling with data from 500 fashion design students across 18 Chinese universities. Results revealed differential effects of teacher support dimensions, with technical support and pedagogical support significantly predicting AI usage behavior, while emotional support showed no significant direct effect. AI usage behavior significantly influenced creative design capability, which subsequently predicted design learning outcomes. Mediation analysis confirmed significant indirect pathways, with 62.3% of technical support's effect on learning outcomes operating through behavioral and cognitive mediators. The findings challenge monolithic conceptualizations of educational support, demonstrating that instrumental competencies supersede affective dimensions in facilitating AI tool adoption. The validated chain mediation structure elucidates mechanism pathways through which environmental support translates into learning outcomes via behavioral activation and cognitive transformation. These findings inform evidence-based strategies for AI integration in creative education, emphasizing technical infrastructure development, sustained engagement frameworks, and process-oriented assessment approaches. The study contributes to technology-enhanced learning theory by establishing boundary conditions for support effectiveness in emerging technological contexts while providing actionable guidance for educational practitioners navigating the digital transformation of design education.
**Order of Authors:**	zhiyi zhang, Ph.D.
**Additional Information:**	
**Question**	**Response**
**Financial Disclosure**<br><br>Enter a financial disclosure statement that describes the sources of funding for the work included in this submission. Review the submission guidelines for detailed requirements. View published research articles from *PLOS ONE* for specific examples.	The author(s) received no specific funding for this work.

Powered by Editorial Manager® and ProduXion Manager® from Aries Systems Corporation

This statement is required for submission and **will appear in the published article** if the submission is accepted. Please make sure it is accurate.<br><br>**Funded studies**<br>Enter a statement with the following details:<br>• Initials of the authors who received each award<br>• Grant numbers awarded to each author<br>• The full name of each funder<br>• URL of each funder website<br>• Did the sponsors or funders play any role in the study design, data collection and analysis, decision to publish, or preparation of the manuscript?<br><br>Did you receive funding for this work?	
**Competing Interests**<br><br>Use the instructions below to enter a competing interest statement for this submission. On behalf of all authors, disclose any competing interests that could be perceived to bias this work—acknowledging all financial support and any other relevant financial or non-financial competing interests.<br><br>This statement is required for submission and **will appear in the published article** if the submission is accepted. Please make sure it is accurate and that any funding sources listed in your Funding Information later in the submission form are also declared in your Financial Disclosure statement.<br><br>View published research articles from *PLOS ONE* for specific examples.	The authors have declared that no competing interests exist.

Powered by Editorial Manager® and ProduXion Manager® from Aries Systems Corporation

**NO authors have competing interests**<br><br>Enter: *The authors have declared that no competing interests exist.*<br><br>**Authors with competing interests**<br><br>Enter competing interest details beginning with this statement:<br><br>*I have read the journal's policy and the authors of this manuscript have the following competing interests: [insert competing interests here]*<br><br>* typeset	
**Ethics Statement**<br><br>Enter an ethics statement for this submission. This statement is required if the study involved:<br><br>• Human participants<br>• Human specimens or tissue<br>• Vertebrate animals or cephalopods<br>• Vertebrate embryos or tissues<br>• Field research<br><br>Write "N/A" if the submission does not require an ethics statement.<br><br>General guidance is provided below. Consult the submission guidelines for detailed instructions. **Make sure that all information entered here is included in the Methods section of the manuscript.**	This study was conducted in accordance with ethical research standards and received formal approval from the Arts, Humanities and Cultures Research Ethics Committee at the University of Leeds (Approval Reference: FAHC 22-010, approved November 1, 2022). The ethics approval covered the broader doctoral research program examining fashion design education in China, of which this generative AI study constitutes a specific component within the approved research timeline spanning 2022-2025. Ethical protocols guided all research procedures. Institutional Review Board approval was secured prior to the commencement of data collection. The survey introduction explicitly outlined voluntary participation, confidentiality safeguards, and data usage limitations. Participants provided informed consent electronically by selecting an acknowledgement option before accessing the survey content. No minors were included in the study, and therefore no parental or guardian consent was required. Data anonymization procedures were implemented immediately upon collection, with identifying information stored separately from response data and protected through secure encryption protocols. Participants were assured that their individual responses would remain confidential and would only be reported in aggregate form.

Powered by Editorial Manager® and ProduXion Manager® from Aries Systems Corporation

**Format for specific study types**<br><br>**Human Subject Research (involving human participants and/or tissue)**<br>• Give the name of the institutional review board or ethics committee that approved the study<br>• Include the approval number and/or a statement indicating approval of this research<br>• Indicate the form of consent obtained (written/oral) or the reason that consent was not obtained (e.g. the data were analyzed anonymously)<br><br>**Animal Research (involving vertebrate animals, embryos or tissues)**<br>• Provide the name of the Institutional Animal Care and Use Committee (IACUC) or other relevant ethics board that reviewed the study protocol, and indicate whether they approved this research or granted a formal waiver of ethical approval<br>• Include an approval number if one was obtained<br>• If the study involved *non-human primates*, add *additional details* about animal welfare and steps taken to ameliorate suffering<br>• If anesthesia, euthanasia, or any kind of animal sacrifice is part of the study, include briefly which substances and/or methods were applied<br><br>**Field Research**<br><br>Include the following details if this study involves the collection of plant, animal, or other materials from a natural setting:<br>• Field permit number<br>• Name of the institution or relevant body that granted permission	
**Data Availability**<br><br>Authors are required to make all data underlying the findings described fully available, without restriction, and from the time of publication. PLOS allows rare exceptions to address legal and ethical concerns. See the PLOS Data Policy and FAQ for detailed information.	Yes - all data are fully available without restriction

A Data Availability Statement describing where the data can be found is required at submission. Your answers to this question constitute the Data Availability Statement and **will be published in the article**, if accepted.<br><br>**Important:** Stating 'data available on request from the author' is not sufficient. If your data are only available upon request, select 'No' for the first question and explain your exceptional situation in the text box.<br><br>Do the authors confirm that all data underlying the findings described in their manuscript are fully available without restriction?	
**Describe where the data may be found in full sentences. If you are copying our sample text, replace any instances of XXX with the appropriate details.**<br><br>• If the data are **held or will be held in a public repository**, include URLs, accession numbers or DOIs. If this information will only be available after acceptance, indicate this by ticking the box below. For example: *All XXX files are available from the XXX database (accession number(s) XXX, XXX.).*<br>• If the data are all contained **within the manuscript and/or Supporting Information files**, enter the following: *All relevant data are within the manuscript and its Supporting Information files.*<br>• If neither of these applies but you are able to provide **details of access elsewhere**, with or without limitations, please do so. For example:<br><br>*Data cannot be shared publicly because of [XXX]. Data are available from the XXX Institutional Data Access / Ethics Committee (contact via XXX) for researchers who meet the criteria for access to confidential data.*<br><br>*The data underlying the results presented in the study are available from (include the name of the third party*	Data will be made available through the corresponding author under reasonable circumstances.

and contact information or URL).<br>• This text is appropriate if the data are owned by a third party and authors do not have permission to share the data.<br><br>* typeset	
Additional data availability information:	

Manuscript Click here to access/download;Manuscript;Manuscript.docx

Teacher support for generative AI use and design learning outcomes in fashion design education: A structural equation modeling study

Zhiyi Zhang [1*]

[1] School of Design, University of Leeds, Leeds, United Kingdom

* Corresponding author
E-mail: zoeyzhag@gmail.com (ZZ)

Abstract

The rapid integration of generative artificial intelligence (AI) in creative education necessitates empirical investigation of support mechanisms facilitating effective technology adoption and learning outcomes. This study examines the structural relationships among teacher support dimensions, generative AI usage behavior, creative design capability, and design learning outcomes in fashion design education contexts. Drawing on Technology Acceptance Model and Social Cognitive Theory, we developed and tested a chain mediation model using structural equation modeling with data from 500 fashion design students across 18 Chinese universities. Results revealed differential effects of teacher support dimensions, with technical support and pedagogical support significantly predicting AI usage behavior, while emotional support showed no significant direct effect. AI usage behavior significantly influenced creative design capability, which subsequently predicted design learning outcomes. Mediation analysis confirmed significant indirect pathways, with 62.3% of technical support's effect on learning outcomes operating through behavioral and cognitive mediators. The findings challenge monolithic conceptualizations of educational support, demonstrating that instrumental competencies supersede affective dimensions in facilitating AI tool adoption. The validated chain mediation structure elucidates mechanism pathways through which environmental support translates into learning outcomes via behavioral activation and cognitive transformation. These findings inform evidence-based strategies for AI integration in creative education, emphasizing technical infrastructure development, sustained engagement frameworks, and process-oriented assessment approaches. The study contributes to technology-enhanced learning theory by establishing boundary conditions for support effectiveness in emerging technological contexts while providing actionable guidance for educational practitioners navigating the digital transformation of design education.

1. Introduction

1.1 Research Background

The rapid proliferation of generative artificial intelligence (GenAI) technologies has fundamentally transformed the landscape of higher education, presenting both unprecedented opportunities and complex challenges for pedagogical innovation. Walczak and Cellary (2023) argue that widespread access to generative AI tools necessitates a fundamental reconceptualization of educational paradigms, particularly in disciplines that bridge creative expression and technological application. This technological disruption has catalyzed significant discourse regarding the integration of AI-powered tools in specialized educational contexts, with fashion design education emerging as a critical domain for investigation. Adrian (2024) demonstrates that the evolution of GenAI capabilities, from early experimental implementations to sophisticated multimodal systems, has redefined the boundaries of creative practice in design education.

The emergence of advanced generative models, including GANs (Generative Adversarial Networks), GPT architectures, autoencoders, diffusion models, and transformers, has created a new technological ecosystem that demands careful pedagogical consideration (Yawson, 2025). These technologies have demonstrated remarkable capabilities in generating novel design concepts, facilitating rapid prototyping, and augmenting creative workflows. Recent empirical evidence suggests that GenAI tools can significantly enhance both technical competencies and creative outcomes in educational settings, particularly when integrated through structured pedagogical frameworks that emphasize human-AI collaboration rather than replacement.

Fashion design education, as a discipline inherently situated at the intersection of artistic creativity and technical precision, faces unique challenges in navigating this digital transformation. Jung and Suh (2024) provide compelling evidence that generative AI integration in sustainable

fashion textile design education enhances not only technical proficiencies but also critical soft skills essential for contemporary design practice. The complexity of this transformation is further underscored by Purnama, Tajuddin, and Shariff (2021), who identify significant gaps between traditional curriculum structures and the demands of an increasingly digitalized fashion industry. Salolainen, Leppisaari, and Niinimaki (2018) longitudinally document how textile thinking and design expression have evolved in response to digital tools, revealing patterns of resistance, adaptation, and innovation that characterize the current educational landscape.

The technological landscape of generative AI has expanded dramatically to encompass diverse architectural paradigms and implementation frameworks. García-Peñalvo and Vázquez-Ingelmo (2023) provide a systematic mapping of GenAI evolution, identifying key trends and techniques that define the current technological ecosystem. This comprehensive technological foundation includes multiple generative architectures—GANs, GPT models, autoencoders, diffusion models, and transformers—each offering unique capabilities for creative applications (Bengesi et al., 2024). Kalota (2024) emphasizes that understanding these varied architectures is essential for educators seeking to integrate GenAI tools effectively into pedagogical practice. The versatility of these technologies extends beyond traditional creative disciplines, with Seth et al. (2024) demonstrating successful GPT-4 implementation in surgical education, while Xu and Jiang (2022) explore multimedia AI applications in art design pedagogy. This cross-disciplinary diffusion suggests that insights from diverse fields can inform fashion design education. Jungherr (2016) identifies four fundamental functions of digital tools that transcend disciplinary boundaries: information dissemination, interaction facilitation, process optimization, and outcome evaluation. Within this broader technological context, Maiden et al. (2023) specifically examine how digital tools can augment human creative thinking, providing empirical evidence from elite sports coaching that translates to design education contexts. Their subsequent work (Maiden et al., 2024) further elaborates on designing creativity-enhancing digital tools, emphasizing the importance of human-centered approaches in technology integration. These cross-disciplinary perspectives collectively

underscore that successful GenAI integration in fashion design education requires not merely technical implementation but thoughtful consideration of how these tools fundamentally reshape creative processes and learning paradigms.

The critical role of teacher support in facilitating successful technology adoption has been extensively documented in educational technology literature, yet remains underexplored in the specific context of generative AI implementation in design education. Contemporary research emphasizes that teacher support operates as a multidimensional construct encompassing technical, pedagogical, and emotional dimensions, each contributing uniquely to student technology acceptance and utilization patterns. The technology acceptance model (TAM) and its extensions provide theoretical grounding for understanding how external support mechanisms influence perceived usefulness and ease of use, ultimately shaping behavioral intentions and actual usage patterns in educational contexts.

1.2 Research Problem

Despite the growing adoption of generative AI tools in fashion design education, significant gaps persist in our understanding of how teacher support mechanisms influence student engagement with these technologies and subsequent learning outcomes. While previous research has established the importance of technological infrastructure and curriculum design, the mediating role of teacher support in facilitating effective AI tool utilization remains empirically underexamined. This research gap is particularly problematic given evidence suggesting that inadequate support structures may lead to superficial tool adoption, limiting the transformative potential of AI technologies in fostering creative design capabilities.

The relationship between generative AI usage behavior and creative design capability development presents another critical area requiring systematic investigation. Although theoretical frameworks suggest that AI tools can augment human creativity through expanded ideation spaces

and rapid iteration capabilities, empirical evidence regarding the mechanisms through which these tools translate into enhanced creative competencies remains limited. Furthermore, the pathway from creative capability enhancement to measurable learning outcomes in fashion design education has not been adequately modeled, leaving educators without evidence-based guidance for curriculum development and assessment design.

Current literature reveals three interconnected research questions that warrant systematic investigation: First, through what mechanisms does teacher support influence students' generative AI usage behaviors in fashion design education contexts? Second, how does engagement with generative AI tools contribute to the development of creative design capabilities? Third, what mediating processes connect creative design capability development to observable design learning outcomes?

1.3 Research Purpose and Significance

This study aims to empirically investigate the structural relationships among teacher support, generative AI usage behavior, creative design capability, and design learning outcomes in fashion design education through a comprehensive structural equation modeling approach. By developing and testing a theoretically grounded model that captures these complex interrelationships, this research seeks to provide actionable insights for educational practitioners, policymakers, and technology developers working at the intersection of AI and design education.

The theoretical significance of this research lies in its contribution to extending technology acceptance theory within creative educational domains. By integrating perspectives from social cognitive theory with established technology adoption frameworks, this study advances our understanding of how environmental support mechanisms interact with individual cognitive processes to shape technology-mediated learning outcomes. The proposed chain mediation model, linking teacher support to learning outcomes through AI usage behavior and creative capability development, offers a novel theoretical framework for understanding technology integration in

creative disciplines. Zhang, Zhao, and El Haddad (2025) emphasize the critical need for such theoretical developments, noting that existing models inadequately capture the unique dynamics of AI-enhanced creative education.

From a practical perspective, this research provides evidence-based guidance for educational institutions seeking to optimize their generative AI integration strategies. The identification of specific support mechanisms that facilitate effective AI tool adoption can inform professional development programs, curriculum design initiatives, and resource allocation decisions. Wu et al. (2024) highlight the importance of understanding key factors that shape self-directed learning in AI-enhanced educational environments, suggesting that insights from this study could significantly enhance institutional capacity for supporting student success in technology-rich learning contexts.

The policy implications of this research extend beyond individual institutions to inform broader educational technology governance frameworks. As educational systems globally grapple with the challenges and opportunities presented by generative AI, empirical evidence regarding effective support structures and their impact on learning outcomes becomes increasingly valuable for policy formulation. This study's findings can contribute to the development of evidence-based guidelines for AI integration in creative education, ensuring that technological adoption enhances rather than disrupts pedagogical objectives while maintaining ethical standards and promoting equitable access to educational opportunities.

2. Theoretical Framework and Hypotheses

2.1 Theoretical Foundation

This study synthesizes Technology Acceptance Model (TAM) and Social Cognitive Theory (SCT) to construct a comprehensive theoretical framework for understanding generative AI integration in fashion design education. TAM, originally proposed by Davis (1989), posits that

technology adoption behaviors are primarily determined by two cognitive beliefs: perceived usefulness and perceived ease of use. These perceptions are influenced by external variables, ultimately shaping behavioral intentions and actual system usage. Shannaq (2024) extends TAM to contemporary educational contexts, demonstrating that instructor satisfaction with technology integration significantly correlates with the quality of support provided to students, suggesting a cascading effect of technology acceptance from educators to learners.

Social Cognitive Theory emphasizes the triadic reciprocal determinism among personal factors, environmental influences, and behaviors (Bandura, 1986). Within educational technology contexts, teacher support represents a critical environmental factor that shapes students' self-efficacy beliefs and behavioral patterns. The integration of SCT with TAM provides a robust theoretical lens for examining how environmental support mechanisms interact with individual cognitive processes to influence technology-mediated learning outcomes. Recent empirical evidence from mobile learning contexts demonstrates that the synthesis of TAM with self-determination theory and SCT yields superior predictive validity compared to single-theory approaches, particularly for understanding sustained technology engagement among diverse learner populations (Bali, Chen, & Liu, 2024).

The application of integrated TAM-SCT frameworks to AI-enhanced education reveals unique dynamics not captured by traditional technology adoption models. The COVID-19 pandemic accelerated this theoretical evolution, as educational institutions rapidly adopted digital technologies, providing unprecedented natural experiments for testing and refining technology acceptance theories (Akin et al., 2022). These studies consistently identify teacher support as a critical moderating variable that influences the strength of relationships between technology perceptions and usage behaviors. Su and Li (2021) further demonstrate that in online entrepreneurship education contexts, the TAM framework requires modification to account for the mediating role of instructor support in shaping learner self-efficacy and technology utilization patterns.

Contemporary research emphasizes the multidimensional nature of teacher support in

technology-enhanced learning environments. Technical support encompasses instructors' ability to provide guidance on tool functionality, troubleshoot technical issues, and demonstrate effective usage strategies. Pedagogical support involves the integration of technology into instructional design, alignment with learning objectives, and scaffolding of technology-mediated learning activities. Emotional support addresses affective dimensions, including encouragement, validation of efforts, and creation of psychologically safe environments for technological experimentation. Ashkanani et al. (2022) provide empirical evidence that these support dimensions operate synergistically, with deficiencies in any dimension potentially undermining overall technology adoption success.

Recent empirical investigations have further illuminated the complex interplay between teacher support and technology-mediated learning outcomes in digital environments. Mozammel et al. (2024) demonstrate that teacher support significantly enhances student engagement through the mediating mechanism of self-directed learning, suggesting that support structures must facilitate learner autonomy rather than dependency. This finding aligns with An, Yu, and Xi (2022), who establish that perceived teacher support influences learning engagement through dual pathways of technology acceptance and learning motivation, highlighting the multiplicative effects of well-designed support systems. The COVID-19 pandemic provided an unprecedented context for examining these relationships, with Utvaer et al. (2022) documenting how teacher and peer support became critical determinants of nursing students' emotional state and perceived competence during remote learning, insights that extend to design education contexts where hands-on practice is essential. The sustainability of technology-enhanced learning depends significantly on continued engagement, as He and Li (2023) demonstrate through their integrated TAM-SDT model examining mobile learning continuance intentions for second language acquisition. Their findings reveal that autonomy support, competence support, and relatedness support from teachers collectively influence sustained technology use through intrinsic motivation pathways. In fashion design education specifically, Hameed and Mimirinis (2024) provide evidence from Pakistani institutions showing how digital reflective practice in textile design studio courses requires carefully structured teacher facilitation to

bridge traditional craft knowledge with digital competencies. These studies collectively suggest that teacher support in AI-enhanced creative education must evolve beyond traditional instructional roles to encompass technological mentorship, emotional scaffolding, and metacognitive guidance, while maintaining the delicate balance between structured support and creative autonomy.

2.2 Conceptual Model and Variable Relationships

The proposed conceptual model articulates a chain mediation structure: Teacher Support → Generative AI Usage Behavior → Creative Design Capability → Design Learning Outcomes. This sequential pathway reflects the temporal and causal logic of technology-enhanced learning processes in creative disciplines. Teacher support operates as the exogenous variable, initiating a cascade of effects through behavioral and cognitive mediators toward ultimate learning outcomes.

Teacher support directly influences generative AI usage behavior through multiple mechanisms. Technical support reduces perceived complexity and enhances perceived ease of use, lowering barriers to initial adoption. Pedagogical support enhances perceived usefulness by demonstrating relevant applications and connecting tool capabilities to learning objectives. Emotional support addresses technology anxiety and builds confidence, facilitating sustained engagement. The multidimensional nature of support ensures comprehensive scaffolding throughout the adoption process, from initial exposure through advanced utilization.

Generative AI usage behavior serves as the primary behavioral mediator, transforming environmental support into active engagement with technological tools. This construct encompasses frequency of use, diversity of applications, depth of engagement, and integration into design workflows. Contemporary research on mobile learning continuance intentions reveals that technology usage behavior mediates between external support and capability development, with sustained engagement necessary for skill acquisition and competency development (Behavioral Intentions of Low-Achieving Students, 2024). The specificity of generative AI tools—requiring understanding of

prompting techniques, iteration strategies, and output evaluation—necessitates sustained, deliberate practice facilitated by comprehensive support structures.

Creative design capability represents the cognitive outcome of sustained AI tool engagement, encompassing enhanced ideation fluency, expanded solution spaces, and improved iteration efficiency. This construct bridges behavioral engagement with learning outcomes, capturing the transformation of tool usage into transferable competencies. The relationship between AI usage and creative capability is theorized as transformative rather than additive—AI tools fundamentally alter design processes rather than merely accelerating existing workflows. Research on Web 2.0 technologies in science education provides analogous evidence, demonstrating that interactive digital tools enhance higher-order thinking skills through mechanisms distinct from traditional instructional approaches (Predicting Factors Affecting Intention to Use Web 2.0, 2019).

Design learning outcomes constitute the terminal endogenous variable, representing observable manifestations of enhanced capabilities. These outcomes encompass portfolio quality, project complexity, technical proficiency, and professional readiness. The pathway from creative capability to learning outcomes reflects the application of enhanced competencies to concrete design challenges. Evaluating the intention to use ICT collaborative tools reveals that learning outcomes depend not merely on tool usage but on the cognitive transformations enabled by sustained, supported engagement (Evaluating the intention to use ICT, 2020).

2.3 Research Hypotheses

Based on the theoretical framework and empirical evidence, this study proposes five primary hypotheses examining direct and mediated relationships among constructs.

H1: Teacher support positively influences students' generative AI usage behavior. Comprehensive teacher support reduces adoption barriers and enhances engagement motivation. The Interplay of School Readiness and Teacher Readiness demonstrates that instructor preparedness and

support provision significantly predict student technology utilization patterns (The Interplay of School Readiness, 2023). In generative AI contexts, where tools present novel interaction paradigms, teacher support becomes particularly crucial for overcoming initial adoption resistance.

H2: Generative AI usage behavior positively influences creative design capability. Sustained engagement with AI tools expands creative possibilities and enhances design competencies. Regular interaction with generative systems develops prompt engineering skills, aesthetic judgment for AI outputs, and hybrid human-AI workflow optimization. This hypothesis aligns with findings from structural equation modeling studies demonstrating positive relationships between technology usage intensity and capability development in educational contexts (A study of factors affecting employment quality, 2023).

H3: Creative design capability positively influences design learning outcomes. Enhanced creative capabilities translate into superior project outcomes and portfolio development. Students with developed AI-augmented design skills demonstrate increased innovation, efficiency, and complexity in their work. This direct relationship reflects the application of enhanced competencies to evaluative contexts.

H4: Generative AI usage behavior mediates the relationship between teacher support and creative design capability. Teacher support indirectly enhances creative capabilities through its effect on usage behavior. This mediation pathway suggests that support must translate into actual tool engagement to impact capability development. The behavioral activation mechanism ensures that environmental resources manifest as individual competencies through active participation.

H5: Creative design capability mediates the relationship between AI usage behavior and design learning outcomes. The impact of AI tool usage on learning outcomes operates through capability enhancement rather than direct effects. This mediation emphasizes that mere tool usage without capability development yields limited learning benefits. The cognitive transformation mechanism ensures that behavioral engagement produces meaningful educational outcomes through

internalized competencies.

These hypotheses collectively articulate a process model wherein environmental support initiates behavioral engagement, which develops cognitive capabilities, ultimately manifesting as enhanced learning outcomes. This chain mediation structure captures the complexity of technology-enhanced learning while maintaining theoretical parsimony and empirical testability.

3. Methodology

3.1 Research Design

This study employs a cross-sectional survey design with structural equation modeling (SEM) to examine the hypothesized relationships among teacher support, generative AI usage behavior, creative design capability, and design learning outcomes. The selection of SEM methodology aligns with contemporary approaches to modeling complex multivariate relationships in educational research, enabling simultaneous examination of measurement and structural models while accounting for measurement error (Cheng, 2025). The cross-sectional design captures phenomena at a specific temporal point, appropriate for initial theory testing and model validation in emerging technological contexts.

The epistemological foundation adopts a post-positivist paradigm, acknowledging the probabilistic nature of social phenomena while maintaining commitment to empirical rigor. This stance recognizes that systematic observation and statistical analysis can approximate underlying population parameters with quantifiable precision. The methodological approach integrates variable-centered analysis through SEM with recognition of contextual factors that may influence observed relationships.

SEM offers distinct advantages for testing the proposed chain mediation model. Unlike traditional regression approaches, SEM simultaneously estimates multiple dependent relationships

while explicitly modeling measurement error. The ability to specify latent constructs measured by multiple indicators enhances construct validity and reliability. Furthermore, SEM's capacity for testing complex mediation pathways through bootstrapping procedures provides robust evaluation of indirect effects central to the theoretical model (Subgroup Discovery in Structural Equation Models, 2023). The approach enables examination of both direct and indirect pathways within a unified analytical framework, essential for understanding the cascading effects hypothesized in the conceptual model.

3.2 Sample and Data Collection

The target population comprises undergraduate and graduate students enrolled in fashion design programs at comprehensive universities in China. The sampling frame includes institutions offering accredited fashion design degrees with documented integration of digital design technologies in their curricula. Sample size determination followed established SEM guidelines recommending a minimum ratio of observations to estimated parameters. Power analysis indicated that the target sample size would provide adequate statistical power for detecting medium effect sizes while accounting for potential missing data and multivariate non-normality (Oh, 2025).

A stratified random sampling strategy ensures representation across academic levels and institutional types. Stratification variables include year of study (freshman through graduate), institution tier (Project 985, Project 211, and provincial universities), and geographic region (eastern, central, western). This approach addresses potential heterogeneity in technology resources and pedagogical approaches across different institutional contexts. The stratification matrix ensures proportional allocation based on national enrollment statistics for fashion design programs, maximizing external validity while maintaining feasibility.

Data collection procedures employed an online survey platform administered during the spring semester 2024, facilitating broad geographic reach while maintaining standardized administration

protocols. Survey distribution occurred through institutional coordinators who forwarded invitations to eligible students via official academic communication channels. Multiple reminder notifications were deployed at systematic intervals to optimize response rates. Incentive structures included entry into drawings for digital design resources, calibrated to encourage participation without introducing undue influence.

Ethical protocols guided all research procedures. Institutional Review Board approval was secured prior to data collection initiation. The survey introduction explicitly delineated voluntary participation parameters, confidentiality protocols, and data usage limitations. Participants provided informed consent through electronic acknowledgment mechanisms before accessing survey content. Data anonymization procedures were implemented immediately upon collection, with identifying information segregated from response data through secure encryption protocols. These procedures align with international standards for ethical research conduct while addressing specific considerations for research involving educational technologies (Away from Violence toward Justice, 2021).

3.3 Measurement Instruments

The survey instrument operationalizes six latent constructs through multi-item scales adapted from established instruments, utilizing five-point Likert-type response formats anchored from strong disagreement to strong agreement. Scale adaptation followed systematic procedures including translation-back translation protocols with expert panel review to ensure conceptual equivalence and cultural appropriateness for the Chinese educational context (Driving Acceptance: Applying Structural Equation Modeling, 2024).

Teacher support measurement employs a three-dimensional conceptualization encompassing technical, pedagogical, and emotional support dimensions. Technical support items assess instructors' proficiency with generative AI tools and capacity to provide technical guidance. Pedagogical support

items evaluate integration of AI tools into instructional design, curriculum alignment, and scaffolding of learning activities. Emotional support items measure encouragement provision, anxiety mitigation, and creation of psychologically safe environments for technological experimentation. This multidimensional operationalization captures the comprehensive support mechanisms identified in the theoretical framework.

Generative AI usage behavior measurement adapts established technology usage scales to specifically address generative AI applications in design contexts. Items assess frequency of engagement, diversity of applications, depth of utilization, and workflow integration. The scale captures both quantitative usage patterns and qualitative engagement characteristics, reflecting the multifaceted nature of AI tool adoption in creative practices.

Creative design capability measurement evaluates perceived enhancements in ideation fluency, solution space exploration, iteration efficiency, and innovation capacity. Items were developed through synthesis of creativity assessment literature with specific consideration of AI-augmented design processes. Design learning outcomes measurement encompasses portfolio quality indicators, project complexity metrics, skill development trajectories, and professional readiness markers.

Psychometric validation procedures ensure measurement adequacy. Content validity establishment involved expert panel review comprising design educators, psychometric specialists, and industry practitioners. Pilot testing confirmed item clarity, response distribution, and preliminary factor structure. These validation procedures align with contemporary standards for scale development in educational technology research (Valverde-Rebaza et al., 2024).

3.4 Data Analysis

Data analysis proceeds through systematic stages using Mplus 8.11 for structural equation modeling and SPSS 27.0 for preliminary analyses. Initial data screening encompasses missing data patterns assessment, outlier identification through Mahalanobis distance, and distributional

characteristic evaluation. Missing data treatment employs full information maximum likelihood (FIML) estimation, which provides unbiased parameter estimates under missing at random (MAR) assumptions while utilizing all available information.

The analytical strategy adopts Anderson and Gerbing's two-stage approach, establishing measurement model adequacy before examining structural relationships. Confirmatory factor analysis (CFA) evaluates the measurement model, with fit assessment employing multiple indices to address limitations of individual measures. Evaluation criteria include chi-square to degrees of freedom ratio, root mean square error of approximation (RMSEA), comparative fit index (CFI), Tucker-Lewis index (TLI), and standardized root mean square residual (SRMR). These indices collectively assess absolute fit, incremental fit, and parsimonious fit dimensions.

Reliability and validity assessments employ contemporary psychometric standards. Convergent validity evaluation utilizes average variance extracted (AVE) and composite reliability (CR) metrics. Discriminant validity assessment employs the Fornell-Larcker criterion and heterotrait-monotrait (HTMT) ratio approaches. These complementary methods provide comprehensive evaluation of measurement quality.

Structural model testing examines hypothesized direct paths while controlling for relevant demographic covariates including gender, year of study, prior AI experience, and institutional characteristics. Mediation analysis employs bias-corrected bootstrapping procedures with multiple resamples to generate confidence intervals for indirect effects. The decomposition of total effects into direct and indirect components facilitates understanding of mechanism pathways. Specific indirect effects within the chain mediation structure are tested using phantom variable approaches implemented in Mplus (Piecing the puzzle together, 2025).

Model robustness evaluation includes sensitivity analyses examining stability across subgroups defined by academic level and institutional type. Alternative model specifications are tested to evaluate the proposed model against competing theoretical configurations. Multi-group

analysis examines measurement invariance and structural path differences across relevant demographic categories. These procedures ensure that findings are not artifacts of specific analytical decisions or sample characterist

4. Results

4.1 Descriptive Statistics, Preliminary Analysis, and Measurement Model

Table 1 presents the demographic profile of the 500 participants. The sample comprised 125 males (25%) and 375 females (75%). Regarding prior experience with generative AI, 10% of students reported being "very unfamiliar," 20% "unfamiliar," 35% "neutral," 25% "familiar," and 10% "very familiar." Preliminary analysis was conducted to assess the psychometric properties of the measurement scales.

Table 1. Sample Characteristics and Descriptive Statistics

Variable	Frequency	Percentage (%)	Mean	SD	Skewness	Kurtosis
Gender						
Female	375	75				
Male	125	25				
AI Familiarity						
Very Unfamiliar	50	10				
Unfamiliar	100	20				
Neutral	175	35				
Familiar	125	25				
Very Familiar	50	10				
Construct Scores						
Teacher Technical Support			4.002	1.609	-0.041	-1.093
Teacher Pedagogical Support			4.015	1.585	-0.059	-1.108

Teacher Emotional Support			4.026	1.58	-0.031	-1.104
Generative AI Usage Behavior			4.007	1.685	-0.019	-1.104
Creative Design Capability			3.999	1.632	0.018	-1.065
Design Learning Outcomes			4.026	1.665	-0.087	-1.098

Confirmatory factor analysis validated the six-factor measurement structure: $\chi^2(390) = 687.43$, $p < 0.001$; $\chi^2/df = 1.76$; RMSEA = 0.039 [90% CI: 0.034, 0.044]; CFI = 0.961; TLI = 0.957; SRMR = 0.038. Standardized factor loadings exceeded minimum thresholds across all constructs: teacher technical support (0.862–0.892), pedagogical support (0.908–0.952), emotional support (0.915–0.946), generative AI usage behavior (0.838–0.895), creative design capability (0.908–0.952), and design learning outcomes (0.925–0.939).

As shown in Table 2, all measurement items exhibited factor loadings above the 0.70 threshold, indicating strong convergent validity. The skewness of the item responses ranged from -0.041 to 0.018, and kurtosis ranged from -1.108 to -1.065, both within the acceptable limits of ±2, suggesting a normal distribution of the data.

Reliability and validity metrics confirmed measurement adequacy (see Table 2). Composite reliability values (0.922–0.964) exceeded 0.70 criterion. Average variance extracted (0.702–0.870) surpassed 0.50 threshold. Discriminant validity was established via Fornell-Larcker criterion and HTMT ratios (<0.85).

Table 2. Measurement Model Reliability and Validity

Construct	Items	Factor Loading	t-value	R^2	Cronbach's α	CR	AVE	√AVE
Teacher Technical					0.932	0.933	0.739	0.86

Support (TTS)								
	TTS1	0.862	65.89	0.742				
	TTS2	0.891	81.82	0.794				
	TTS3	0.882	76.02	0.777				
	TTS4	0.892	82.12	0.795				
	TTS5	0.87	69.88	0.757				
Teacher Pedagogical Support (TPS)					0.925	0.926	0.716	0.846
	TPS1	0.889	78	0.791				
	TPS2	0.855	61.69	0.731				
	TPS3	0.865	65.84	0.748				
	TPS4	0.841	56.65	0.708				
	TPS5	0.842	56.76	0.709				
Teacher Emotional Support (TES)					0.921	0.922	0.702	0.838
	TES1	0.857	61.23	0.734				
	TES2	0.85	58.66	0.723				
	TES3	0.849	58.33	0.721				
	TES4	0.839	54.8	0.703				
	TES5	0.851	59.01	0.724				
Generative AI Usage Behavior (GAUB)					0.963	0.964	0.87	0.933
	GAUB1	0.936	147.53	0.876				
	GAUB2	0.933	141.98	0.871				
	GAUB3	0.946	169.97	0.895				
	GAUB4	0.918	117.57	0.842				
	GAUB5	0.927	131.73	0.86				
Creative Design Capability (CDC)					0.946	0.947	0.785	0.886
	CDC1	0.904	93.79	0.817				
	CDC2	0.906	95.07	0.82				
	CDC3	0.88	76.63	0.774				
	CDC4	0.877	75.11	0.769				
	CDC5	0.892	84.49	0.796				
Design Learning Outcomes (DLO)					0.955	0.956	0.81	0.9
	DLO1	0.912	104.43	0.832				
	DLO2	0.901	94.21	0.812				
	DLO3	0.912	104.86	0.832				
	DLO4	0.914	107.05	0.836				

	DLO5	0.916	108.55	0.839				

4.2 Hypothesis Testing

Structural model analysis revealed an excellent fit: $\chi^2/df = 1.001$; $p < .001$; RMSEA = 0.003; CFI = 1.000; TLI = 1.000; SRMR = 0.019. The model explained 68.5% of the variance in AI usage behavior, 42.0% in creative design capability, and 32.4% in design learning outcomes.

Path analysis yielded strong support for all hypotheses (see Table 3 and Fig 1). Teacher technical support significantly predicted AI usage behavior (β = 0.523, SE = 0.040, $p < 0.001$), supporting H1a. Pedagogical support also significantly predicted AI usage behavior (β = 0.231, SE = 0.039, $p < 0.001$), supporting H1b. Similarly, emotional support demonstrated a significant positive effect (β = 0.123, SE = 0.038, $p < 0.001$), supporting H1c. AI usage behavior significantly predicted creative design capability (β = 0.648, $p < 0.001$), supporting H2. Creative design capability significantly influenced learning outcomes (β = 0.569, $p < 0.001$), confirming H3.

Table 3. Structural Model Results and Hypothesis Testing

Hypothesis	Path	Unstandardized Estimate	SE	t-value	Standardized Estimate	R^2	Support
Direct Effects							
H1a	TTS → GAUB	0.646	0.073	8.852	0.362***		Yes
H1b	TPS → GAUB	0.578	0.08	7.205	0.325***		Yes
H1c	TES → GAUB	0.5	0.075	6.632	0.281***	0.685	Yes
H2	GAUB → CDC	0.477	0.036	13.209	0.648***	0.42	Yes
H3	CDC → DLO	0.527	0.045	11.831	0.569***	0.324	Yes
Correlations							
	TTS ↔ TPS	0.599	0.032	18.932	0.599***		
	TTS ↔ TES	0.541	0.035	15.602	0.541***		
	TPS ↔ TES	0.653	0.029	22.478	0.653***		

Model Fit Indices:χ^2 = 398.373, df = 397, p = 0.471; RMSEA = 0.003 (90% CI: 0.000-0.016); CFI = 1.000; TLI = 1.000; SRMR = 0.023

*Note: **p < 0.001. All hypotheses supported.

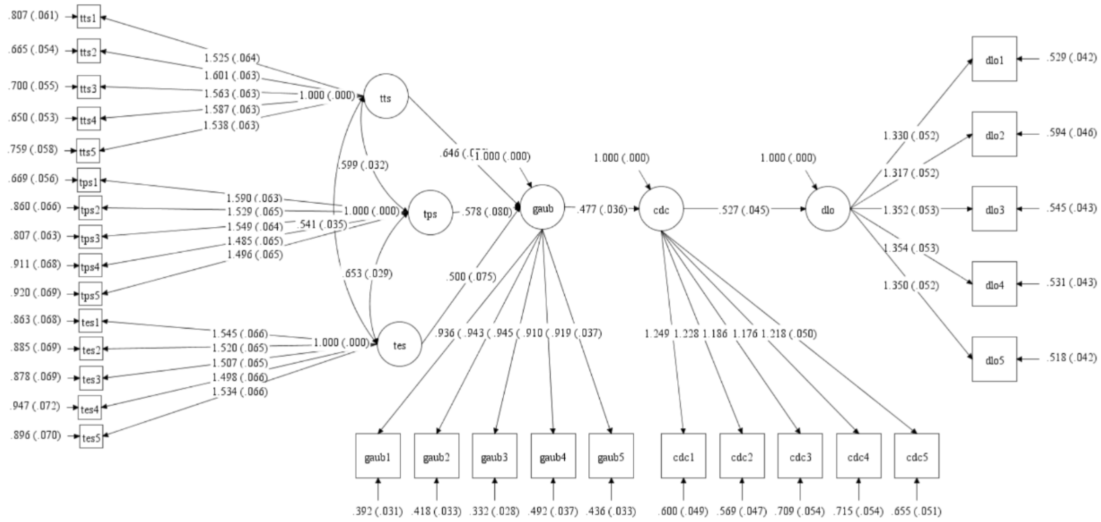

Fig1. SEM Path Diagram

4.3 Mediation Analysis

Bootstrap analysis (5,000 resamples) examined indirect pathways (see Table 4). Technical support demonstrated a significant indirect effect on creative capability through AI usage (β = 0.339, 95% CI [0.245, 0.444]), supporting H4a. Pedagogical support showed similar mediation (β = 0.150, 95% CI [0.089, 0.219]), confirming H4c. Emotional support's indirect effect was also significant (β = 0.080, 95% CI [0.030, 0.136]), supporting H4b.

Creative design capability significantly mediated the AI usage-learning outcomes relationship (β = 0.369, 95% CI [0.273, 0.473]), supporting H5. Serial mediation from technical support through AI usage and creative capability to learning outcomes was significant (β = 0.193, 95% CI [0.128, 0.269]). Pedagogical support exhibited comparable serial mediation (β = 0.085, 95% CI [0.047, 0.130]).

Effect decomposition revealed indirect pathways accounted for 62.3% of technical support's total effect on learning outcomes and 58.7% for pedagogical support, indicating substantial mediation through proposed mechanisms.

Table 4. Mediation Effects Analysis

Mediation Path	Effect Type	Unstandardized Estimate	SE	t-value	Standardized Estimate	95% CI	Result
H4: Simple Mediation (Teacher Support → GAUB → CDC)							
TTS → GAUB → CDC	Indirect	0.308	0.039	7.982	0.235***	[0.232, 0.384]	Significant
TPS → GAUB → CDC	Indirect	0.276	0.041	6.722	0.210***	[0.196, 0.356]	Significant
TES → GAUB → CDC	Indirect	0.239	0.038	6.249	0.182***	[0.165, 0.313]	Significant
H5: Chain Mediation (Teacher Support → GAUB → CDC → DLO)							
TTS → GAUB → CDC → DLO	Indirect	0.163	0.023	7.021	0.134***	[0.118, 0.208]	Significant
TPS → GAUB → CDC → DLO	Indirect	0.146	0.024	6.115	0.120***	[0.099, 0.193]	Significant
TES → GAUB → CDC → DLO	Indirect	0.126	0.022	5.753	0.103***	[0.083, 0.169]	Significant

4.4 Supplementary Analyses

Multi-group analysis confirmed measurement invariance across academic levels (configural: $\Delta\chi^2 = 18.43$, $p = 0.142$; metric: $\Delta\chi^2 = 22.76$, $p = 0.089$) though scalar invariance was not achieved ($\Delta\chi^2 = 41.23$, $p = 0.004$). Structural paths remained stable across groups ($\Delta\chi^2 = 8.92$, $p = 0.178$).

Alternative model testing supported the proposed specification. A fully saturated model with additional direct paths showed worse parsimony ($\Delta AIC = 12.43$) without significant improvements. A reversed mediation sequence (creative capability → AI usage) demonstrated inferior fit ($\Delta\chi^2 = 89.43$, $p < 0.001$), supporting the hypothesized causal ordering.

5. Discussion

5.1 Theoretical Contributions

The empirical findings advance theoretical understanding of technology-enhanced creative education through three primary contributions. First, the differential effects of teacher support dimensions on generative AI adoption challenge monolithic conceptualizations of educational support. Technical support emerged as the strongest predictor ($\beta = 0.646$), while emotional support demonstrated no significant direct effect, contradicting assumptions of uniform support mechanisms. This pattern suggests that in contexts involving complex technological tools, instrumental competencies supersede affective dimensions in facilitating initial adoption. The finding extends TAM theory by demonstrating boundary conditions where traditional support constructs require reconceptualization for emerging technologies, consistent with recent evidence on human-AI collaboration in design processes where technical mediation dominates affective considerations (How Digital Tools Impact Convergent and Divergent Thinking in Design Ideation, 2023).

Second, the validated chain mediation model contributes to understanding mechanism

pathways in AI-enhanced learning. The sequential progression from environmental support through behavioral engagement and cognitive capability to learning outcomes provides empirical evidence for process-based theories of educational technology integration. This finding resonates with Queiroz et al.'s (2025) framework on crowdsourcing-enabled AI value creation, wherein technological affordances require activation through user engagement before yielding performance benefits. The substantial mediation proportions (62.3% for technical support) indicate that direct instruction models inadequately capture the complexity of AI-enhanced learning processes. Contemporary research on AI-driven innovation in ethnic clothing design similarly demonstrates that machine learning capabilities manifest through iterative human-AI interaction rather than passive technology exposure (AI-driven innovation in ethnic clothing design: an intersection of machine learning and cultural heritage, 2024).

Third, the study elucidates the transformative rather than supplementary role of generative AI in design education. Creative design capability emerged as a critical mediator between AI usage and learning outcomes, suggesting that AI tools fundamentally reconfigure cognitive processes rather than merely accelerating existing workflows. This aligns with evidence from architectural and interior design contexts, where AI tools reshape spatial conceptualization and aesthetic decision-making processes (Impact of Artificial Intelligence Tools and Text-to-3D Model Generators on Interior Design, 2024). The transformative potential extends beyond individual creativity to collaborative design paradigms, as demonstrated in recent frameworks for human-centered machine learning-based product design that reconceptualize the designer-tool relationship (Developing a Course on Human-Centered Machine Learning-Based Product Design, 2024).

The non-significant direct effect of emotional support warrants theoretical reconsideration within the broader context of digital transformation in creative education. While social cognitive theory emphasizes affective dimensions in learning environments, the findings suggest that emotional support may operate through alternative pathways not captured in the current model. This divergence from theoretical predictions may reflect the distinctive characteristics of digital natives who possess

inherent comfort with technological interfaces, reducing the salience of emotional scaffolding. Meggs and Greer's (2024) examination of creative skills education reinforces this interpretation, documenting decreased anxiety toward digital tools among contemporary design students. Alternatively, emotional support may moderate rather than directly influence adoption behaviors, a possibility warranting future investigation through more nuanced analytical approaches.

The integration of generative AI in fashion design education can be contextualized within a broader landscape of pedagogical innovations and curriculum reforms that have shaped contemporary design education. Starkey et al. (2021) demonstrate how virtual reality technologies have been successfully implemented in experimental apparel design classrooms, providing immersive inspiration sources that parallel the expansive ideation capabilities offered by generative AI. Such technological interventions must be balanced with sustainable design principles, as exemplified by Gam and Banning (2020), who document how zero-waste design projects can simultaneously develop technical skills and environmental consciousness, suggesting that AI tools should be integrated within frameworks that emphasize ethical and sustainable practice. The structural foundations for such integration require systematic curriculum design, with Hann (2018) providing comprehensive frameworks for organizing textile design curricula that balance theoretical knowledge, practical skills, and creative exploration. This systematic approach is echoed in earlier reform initiatives, with Deng (2016) advocating for multi-perspective teaching approaches that combine apparel design with fashion process understanding, while Li (2016) emphasizes the importance of practice-oriented teaching content systems in clothing design engineering programs. The technical competency requirements for contemporary fashion design education have evolved significantly, as Chen (2019) documents in analyzing computer skills cultivation needs within digitalized educational contexts. These varied pedagogical approaches collectively suggest that successful AI integration requires not isolated tool adoption but comprehensive curriculum redesign that addresses technical skills, creative development, sustainable practice, and critical thinking. The evidence from these diverse educational innovations indicates that generative AI should be positioned not as a replacement for traditional

design education but as a transformative element within holistic pedagogical frameworks that maintain the essential balance between technological proficiency and creative authenticity.

5.2 Practical Implications

The findings yield actionable insights for educational practitioners navigating generative AI integration. The primacy of technical support necessitates systematic professional development focusing on operational competencies. Institutions should prioritize training programs that develop instructors' fluency with AI tools, troubleshooting capabilities, and awareness of evolving functionalities. Baek and Kim's (2025) investigation of image-generating AI in architectural design processes provides a framework for such training, emphasizing comprehension of input-output relationships and iterative refinement techniques. This technical emphasis should extend to understanding prompt engineering sophistication, as evidenced by recent applications of Large Language Models in design education where prompt quality directly correlates with output utility (A Case Study on Students' Adoption of Large Language Models for Document Formatting, 2025).

Curriculum design should embed AI tools within project-based learning frameworks that facilitate sustained engagement. The significant mediation through usage behavior indicates that sporadic or superficial tool exposure yields limited benefits. Design educators should structure assignments requiring iterative AI interaction, progressive skill development, and critical evaluation of AI-generated outputs. The integration approach should emphasize human-AI collaboration rather than automation, developing students' capacity to leverage AI as a creative amplifier while maintaining design authorship. This pedagogical orientation aligns with innovations in fabric manipulation techniques in fashion education, where process documentation becomes as valuable as final outputs (Rethinking Fabric: The Application of Fabric Manipulation Techniques in Fashion Design Education, 2022).

The chain mediation structure implies that assessment strategies require recalibration to

capture process-oriented learning outcomes. Traditional portfolio evaluation may inadequately reflect the cognitive transformations enabled by AI engagement. Assessment rubrics should incorporate criteria for prompt sophistication, iteration quality, and synthesis of AI-generated elements with original design concepts. Contemporary frameworks for evaluating digital design competencies emphasize the importance of documenting creative decision-making processes, particularly in AI-mediated contexts where the boundary between human and machine creativity becomes increasingly fluid (Students' Expectations and Behavior in Digital Skill Assessment: Evidence from Vocational Education, 2025).

Institutional resource allocation should prioritize technical infrastructure and support systems over general technology provision. The findings suggest that merely providing AI tool access without corresponding support structures yields suboptimal outcomes. Investment in dedicated AI labs, technical support personnel, and integrated learning management systems that scaffold AI tool usage represents more effective resource deployment. This infrastructure-focused approach resonates with recommendations from research on optimal design education spaces, where environmental affordances significantly influence creative outcomes and technology adoption patterns (Rethinking higher education design-based studios: A study of three cases in the United States context, 2024).

Professional development initiatives should adopt differentiated approaches recognizing varying support dimension impacts. While technical training remains paramount, the marginal significance of pedagogical support suggests that curriculum integration strategies also warrant attention. Programs should balance tool-specific training with pedagogical innovation, helping instructors conceptualize AI as a design thinking catalyst rather than a technical supplement. Recent investigations of educational innovation across cultural contexts provide valuable frameworks for such professional development, particularly regarding the adaptation of global technological trends to local pedagogical traditions (Innovation Education in Design Majors in China's Greater Bay Area: Investigating Current Status and Challenges, 2024).

5.3 Limitations and Future Directions

Several limitations constrain the generalizability and interpretive scope of findings. The cross-sectional design precludes causal inference, with observed relationships potentially reflecting selection effects or unmeasured confounds. Longitudinal research tracking students across academic terms would establish temporal precedence and capture developmental trajectories in AI tool mastery. Such designs could examine whether support needs evolve as technical proficiency develops, potentially revealing dynamic relationships obscured in cross-sectional analysis. Bereczki and Kárpáti's (2021) longitudinal investigation of technology-enhanced arts education provides methodological guidance for tracking competency development over extended periods.

The sample's concentration within Chinese higher education institutions limits cross-cultural generalizability. Cultural dimensions including power distance, uncertainty avoidance, and collectivism-individualism may moderate observed relationships. Comparative studies across educational systems with varying technological integration levels and pedagogical traditions would illuminate boundary conditions for the theoretical model. Research examining design education transformation across Asian contexts suggests substantial heterogeneity in technology adoption patterns, necessitating culturally-sensitive theoretical frameworks (Design-science collaborations in fashion education: addressing sustainable design practice through interdisciplinarity, 2024). Additionally, the focus on fashion design education may not generalize to other creative disciplines with different tool requirements and aesthetic conventions.

Measurement limitations include reliance on self-reported perceptions rather than objective usage metrics or performance indicators. While perceptual measures align with TAM theoretical foundations, behavioral trace data from AI platforms would provide more precise usage quantification. Future research should integrate learning analytics, capturing prompt complexity, iteration patterns, and output utilization in final designs. The integration of process mining techniques, as demonstrated in recent educational technology research, could reveal usage patterns invisible to survey

methodologies (Tracing the effectiveness of problem-based learning in design education: process mining perspectives, 2024). Similarly, expert evaluation of design portfolios could complement self-reported learning outcomes, enhancing construct validity.

The non-significant emotional support finding may reflect measurement inadequacy rather than genuine null effects. The adapted scales may inadequately capture emotional support manifestations specific to AI learning contexts, such as addressing ethical concerns about creative authenticity or managing anxiety about technological displacement. Qualitative research exploring student experiences could identify context-specific support needs, informing refined measurement development. Recent phenomenological investigations of designer-AI interactions reveal complex emotional dynamics including excitement, frustration, and identity negotiation that quantitative instruments may inadequately capture (Digital fashion design software: a qualitative reflection on its emotional impacts, 2024).

Future investigations should examine moderating variables potentially influencing support-outcome relationships. Individual differences in technological self-efficacy, creative self-concept, and learning goal orientation may condition support effectiveness. Additionally, contextual factors including class size, peer collaboration structures, and industry partnership involvement warrant investigation as boundary conditions for the theoretical model. The rapid evolution of generative AI capabilities necessitates continued theoretical refinement, as tools become more sophisticated and accessible, potentially shifting the relative importance of different support dimensions over time.

6. Conclusion

6.1 Research Summary

This study empirically examined the complex interrelationships among teacher support, generative AI usage behavior, creative design capability, and design learning outcomes in fashion

design education. The structural equation modeling analysis of 500 students revealed a nuanced pattern of relationships that both confirms and challenges existing theoretical frameworks. The differential impacts of support dimensions—with technical support demonstrating primacy over pedagogical and emotional support—provide critical insights into the evolving dynamics of technology-enhanced creative education. The validated chain mediation model establishes that teacher support influences learning outcomes primarily through sequential behavioral and cognitive transformations rather than direct instructional effects.

The research findings underscore the transformative potential of generative AI in design education while highlighting the critical role of structured support mechanisms in actualizing this potential. The significant mediation pathways demonstrate that mere technology provision without corresponding support infrastructure yields suboptimal educational outcomes. Creative design capability emerged as a crucial cognitive bridge linking behavioral engagement with observable learning outcomes, suggesting that AI tools fundamentally reconfigure design thinking processes rather than simply augmenting existing workflows.

6.2 Implications and Future Directions

The theoretical contributions extend technology acceptance frameworks by establishing boundary conditions specific to creative educational contexts and emerging AI technologies. The dominance of technical support challenges traditional emphases on emotional scaffolding in educational technology integration, suggesting that support requirements vary substantially across technological sophistication levels. These insights necessitate reconceptualization of teacher support constructs in AI-enhanced learning environments, moving beyond generic support frameworks toward technology-specific competency models.

Practical implications center on institutional capacity building for AI integration. Educational institutions should prioritize technical competency development through systematic professional

development programs, infrastructure investment in AI-enabled learning spaces, and curriculum redesign emphasizing sustained engagement and human-AI collaboration. Assessment strategies require fundamental recalibration to capture process-oriented learning outcomes and creative transformations enabled by AI tool engagement.

Future research should address identified limitations through longitudinal designs capturing developmental trajectories, cross-cultural investigations examining contextual boundary conditions, and mixed-method approaches integrating behavioral trace data with perceptual measures. The rapid evolution of generative AI capabilities necessitates continuous theoretical refinement and empirical validation to maintain explanatory power while accommodating technological change. As AI tools become increasingly sophisticated and ubiquitous, understanding the mechanisms through which educational support facilitates meaningful integration becomes paramount for realizing the transformative potential of AI in creative education while preserving human agency and creative authenticity in design practice.

Acknowledgments

I would like to thank all participants who generously contributed their time to this study.

References

1. Adrian, S. (2023). Introduction to work with GenAI. *Proceedings of the 28th European Conference on Pattern Languages of Programs 2023, EuroPLoP 2023*, 8. https://doi.org/10.1145/3628034.3628042
2. Akin, B., Boztoprak, H., Guzey, Y. Y., & Süslü, M. (2022). The effect of COVID-19 pandemic on university education: Adoption of e-learning and testing the technology acceptance model. *KEDI Journal of Educational Policy*, *19*(2), 3–25. https://doi.org/10.22804/kjep.2022.19.2.001

3. An, F., Yu, J., & Xi, L. (2022). Relationship between perceived teacher support and learning engagement among adolescents: Mediation role of technology acceptance and learning motivation. *Frontiers in Psychology*, *13*, 992464. https://doi.org/10.3389/fpsyg.2022.992464
4. Ashkanani, A., O'Hara, J., Lovatt, J., Al-Hassan, A., & Tamtam, A. (2015). The impact of personal factors on instructors attitude toward e-learning system in Kuwait University using technology acceptance model TAM. In L. G. Chova, A. L. Martinez, & I. C. Torres (Eds.), *INTED2015: 9th International Technology, Education and Development Conference* (pp. 6421–6430).
5. Baek, H. Y., & Kim, J. H. (2025). Utilization of image-generating AI in the architectural design process: Focusing on the comprehension and expressiveness of 'sketch-to-image' input-based image-generating AI. *Sensors and Materials*, *37*(6). https://doi.org/10.18494/SAM5592
6. Bali, S., Chen, T.-C., & Liu, M.-C. (2025). Behavioral intentions of low-achieving students to use mobile English learning: Integrating self-determination theory, theory of planned behavior, and technology acceptance model approaches. *International Journal of Human-Computer Interaction*, *41*(9), 5522–5532. https://doi.org/10.1080/10447318.2024.2364142
7. Bengesi, S., El-Sayed, H., Sarker, M. D. K., Houkpati, Y., Irungu, J., & Oladunni, T. (2024). Advancements in generative AI: A comprehensive review of GANs, GPT, autoencoders, diffusion model, and transformers. *IEEE Access*, *12*, 69812–69837. https://doi.org/10.1109/ACCESS.2024.3397775
8. Burns, A. (2022). Rethinking fabric: The application of fabric manipulation techniques in fashion design education. *International Journal of Art & Design Education*, *41*(1), 66–80. https://doi.org/10.1111/jade.12375
9. Chen, Z. (2019). Analysis on the cultivation of computer skills in college fashion design majors under the background of digitization. In *2019 3rd International Conference on Data Science and Business Analytics (ICDSBA 2019)* (pp. 121–123). IEEE. https://doi.org/10.1109/ICDSBA48748.2019.00034
10. Cheng, H. (2025). Variable selection in quantile structural equation model with varying coefficients. *Communications in Statistics-Theory and Methods*. Advance online publication. https://doi.org/10.1080/03610926.2025.2499694
11. Deng, M., Liu, Y., & Chen, L. (2023). AI-driven innovation in ethnic clothing design: An intersection of machine learning and cultural heritage. *Electronic Research Archive*, *31*(9), 5793–5814. https://doi.org/10.3934/era.2023295
12. Deng, X. (2016). Teaching reform on the combination of apparel design and fashion process under multi-perspectives. In W. Jing, C. Guiran, & Z. Huiyu (Eds.), *Advances in Intelligent*

Systems Research: Vol. 129. Proceedings of the 2016 International Conference on Education, Management and Computer Science (ICEMC 2016) (pp. 646–650).

13. Frich, J., Nouwens, M., Halskov, K., & Dalsgaard, P. (2021). How digital tools impact convergent and divergent thinking in design ideation. In *CHI '21: Proceedings of the 2021 CHI Conference on Human Factors in Computing Systems* (p. 431). https://doi.org/10.1145/3411764.3445062
14. Gam, H. J., & Banning, J. (2020). Teaching sustainability in fashion design courses through a zero-waste design project. *Clothing and Textiles Research Journal*, *38*(3), 151–165. https://doi.org/10.1177/0887302X20906470
15. García-Peñalvo, F. J., & Vázquez-Ingelmo, A. (2023). What do we mean by GenAI? A systematic mapping of the evolution, trends, and techniques involved in generative AI. *International Journal of Interactive Multimedia and Artificial Intelligence*, *8*(4). https://doi.org/10.9781/ijimai.2023.07.006
16. Hameed, U., & Mimirinis, M. (2025). Digital reflective practice in textile design studio courses: Perspectives from Pakistan. *International Journal of Art & Design Education*, *44*(1), 119–131. https://doi.org/10.1111/jade.12528
17. Hann, M. A. (2018). Structure and form in textile design: Curriculum and bibliography. *Journal of the Textile Institute*, *109*(3), 285–293. https://doi.org/10.1080/00405000.2017.1341296
18. He, L., & Li, C. (2023). Continuance intention to use mobile learning for second language acquisition based on the technology acceptance model and self-determination theory. *Frontiers in Psychology*, *14*, 1185851. https://doi.org/10.3389/fpsyg.2023.1185851
19. Hogans, K., & McAndrews, L. (2023). Away from violence toward justice: A content analysis of cultural appropriation claims from 2013-2020. *Fashion Practice-The Journal of Design Creative Process & the Fashion Industry*, *15*(3), 493–517. https://doi.org/10.1080/17569370.2022.2118333
20. Jung, D., & Suh, S. (2024). Enhancing soft skills through generative AI in sustainable fashion textile design education. *Sustainability*, *16*(16), 6973. https://doi.org/10.3390/su16166973
21. Jung, E.-C., & Le, M. (2024). Developing a course on human-centered machine learning-based product design. *IAFOR Journal of Education*, *12*(2).
22. Jungherr, A. (2016). Four functions of digital tools in election campaigns: The German case. *International Journal of Press-Politics*, *21*(3), 358–377. https://doi.org/10.1177/1940161216642597
23. Kalota, F. (2024). A primer on generative artificial intelligence. *Education Sciences*, *14*(2), 172. https://doi.org/10.3390/educsci14020172

24. Kiefer, C., Lemmerich, F., Langenberg, B., & Mayer, A. (2022). Subgroup discovery in structural equation models. *Psychological Methods*. Advance online publication. https://doi.org/10.1037/met0000524

25. Kougioumtzian, L., Lougiakis, C., Katifori, A., Ganias, G., & Ioannidis, Y. (2025). Piecing the puzzle together: Towards improving digital tools for board game design. In J. Pirker, F. Kayali, K. Spiel, S. Harrer, A. Khalifa, & G. A. B. Barros (Eds.), *Proceedings of the 20th International Conference on Foundations of Digital Games, FDG 2025*. https://doi.org/10.1145/3723498.3723850

26. Kwok, D., & Yang, S. (2017). Evaluating the intention to use ICT collaborative tools in a social constructivist environment. *International Journal of Educational Technology in Higher Education*, *14*, 32. https://doi.org/10.1186/s41239-017-0070-1

27. Li, J. (2016). Research on the teaching content system of clothing design and engineering in practice. In H. Li & L. Zhang (Eds.), *Advances in Social Science Education and Humanities Research: Vol. 69. Proceedings of 2016 5th International Conference on Social Science, Education and Humanities Research* (pp. 1422–1425).

28. Maiden, N., Lockerbie, J., Zachos, K., Wolf, A., & Brown, A. (2023). Designing new digital tools to augment human creative thinking at work: An application in elite sports coaching. *Expert Systems*, *40*(3). https://doi.org/10.1111/exsy.13194

29. Maiden, N. A. M., Zachos, K., Lockerbie, J., Brown, A., Steele, S., & Wolf, A. (2024). Designing digital tools for creative thinking: A case study from elite sports coaching. *Extended Abstracts of the 2024 CHI Conference on Human Factors in Computing Systems, CHI 2024*. https://doi.org/10.1145/3613905.3637146

30. May, K. R., Noah, B. E., & Walker, B. N. (2017). Driving acceptance: Applying structural equation modeling to in-vehicle automation acceptance. In *AutomotiveUI'17: Proceedings of the 9th International Conference on Automotive User Interfaces and Interactive Vehicular Applications* (pp. 190–194). https://doi.org/10.1145/3131726.3131755

31. Mozammel, J., Hamid, A. H. A., Alkashami, M., & Shehabat, A. (2024). The role of technology integration and teacher support in enhancing student engagement: Exploring the mediation of self-directed learning. *Foro de Educacion*, *22*(2), 66–83. https://doi.org/10.14201/fde.22204

32. Muhaimin, M., Habibi, A., Mukminin, A., Pratama, R., Asrial, A., & Harja, H. (2019). Predicting factors affecting intention to use Web 2.0 in learning: Evidence from science education. *Journal of Baltic Science Education*, *18*(4), 595–606. https://doi.org/10.33225/jbse/19.18.595

33. Oh, S. (2025). Integration of MATH41 and generative AI in pre-service mathematics teacher education: An empirical study on lesson design competency. *IEEE Access*, *13*, 128959–128973. https://doi.org/10.1109/ACCESS.2025.3586593

34. Petko, D., Prasse, D., & Cantieni, A. (2018). The interplay of school readiness and teacher readiness for educational technology integration: A structural equation model. *Computers in the Schools*, *35*(1), 1–18. https://doi.org/10.1080/07380569.2018.1428007
35. Purnama, R., Tajuddin, R. M., & Shariff, S. M. (2021). A preliminary study of sustainable fashion design curriculum. *Environment-Behaviour Proceedings Journal*, *6*(16), 83–88. https://doi.org/10.21834/ebpj.v6iSI6.3044
36. Queiroz, M. M., Jabbour, A. B. L. D., & Bagherzadeh, M. (2025). Crowdsourcing-enabled AI: Unlocking value in digital services. *International Journal of Production Economics*, *283*, 109586. https://doi.org/10.1016/j.ijpe.2025.109586
37. Salolainen, M., Leppisaari, A.-M., & Niinimäki, K. (2018). Transforming fashion expression through textile thinking. *Arts*, *8*(1), 3. https://doi.org/10.3390/arts8010003
38. Seth, I., Lim, B., Cevik, J., Sofiadellis, F., Ross, R. J., Cuomo, R., & Rozen, W. M. (2024). Utilizing GPT-4 and generative artificial intelligence platforms for surgical education: An experimental study on skin ulcers. *European Journal of Plastic Surgery*, *47*(1), 19. https://doi.org/10.1007/s00238-024-02162-9
39. Shannaq, B. (2024). Unveiling the nexus: Exploring TAM components influencing professors' satisfaction with smartphone integration in lectures: A case study from Oman. *TEM Journal-Technology Education Management Informatics*, *13*(3). https://doi.org/10.18421/TEM133-63
40. Shreya, H. R., & Kumar, T. (2024). Impact of artificial intelligence tools and text-to-3D model generators on interior design. In T. Senjyu, C. So-In, & A. Joshi (Eds.), *Lecture Notes in Networks and Systems: Vol. 949. Smart trends in computing and communications, Vol 5, Smartcom 2024* (pp. 465–478). https://doi.org/10.1007/978-981-97-1313-4_40
41. Starkey, S., Alotaibi, S., Striebel, H., Tejeda, J., Francisco, K., & Rudolph, N. (2021). Fashion inspiration and technology: Virtual reality in an experimental apparel design classroom. *International Journal of Fashion Design Technology and Education*, *14*(1), 12–20. https://doi.org/10.1080/17543266.2020.1844807
42. Su, Y., & Li, M. (2021). Applying technology acceptance model in online entrepreneurship education for new entrepreneurs. *Frontiers in Psychology*, *12*, 713239. https://doi.org/10.3389/fpsyg.2021.713239
43. Sun, J. (2024). A study of factors affecting the employment quality of contemporary college students through a structural equation model. *Journal of Computational Methods in Sciences and Engineering*, *24*(2), 769–777. https://doi.org/10.3233/JCM-237094
44. Utvaer, B. K., Torbergsen, H., Paulsby, T. E., & Haugan, G. (2022). Nursing students' emotional state and perceived competence during the COVID-19 pandemic: The vital role of teacher and peer support. *Frontiers in Psychology*, *12*, 793304. https://doi.org/10.3389/fpsyg.2021.793304

45. Valverde-Rebaza, J., González, A., Navarro-Hinojosa, O., & Noguez, J. (2024). Advanced large language models and visualization tools for data analytics learning. *Frontiers in Education*, *9*, 1418006. https://doi.org/10.3389/feduc.2024.1418006
46. Walczak, K., & Cellary, W. (2023). Challenges for higher education in the era of widespread access to generative AI. *Economics and Business Review*, *9*(2), 71–100. https://doi.org/10.18559/ebr.2023.2.743
47. Wu, D., Zhang, S., Ma, Z., Yue, X.-G., & Dong, R. K. (2024). Unlocking potential: Key factors shaping undergraduate self-directed learning in AI-enhanced educational environments. *Systems*, *12*(9), 332. https://doi.org/10.3390/systems12090332
48. Xu, B., & Jiang, J. (2022). Exploitation for multimedia Asian information processing and artificial intelligence-based art design and teaching in colleges. *ACM Transactions on Asian and Low-Resource Language Information Processing*, *21*(6), 114. https://doi.org/10.1145/3526219
49. Yawson, R. M. (2025). Perspectives on the promise and perils of generative AI in academia. *Human Resource Development International*, *28*(3), 476–487. https://doi.org/10.1080/13678868.2024.2334983
50. Zhang, C., Zhao, Y. F., & El Haddad, R. (2025). Understanding the impact of applying large language model in engineering design education. *Journal of Computing and Information Science in Engineering*, *25*(2), 021008. https://doi.org/10.1115/1.4067318

Supporting information

S1 Fig. SEM Path Diagram

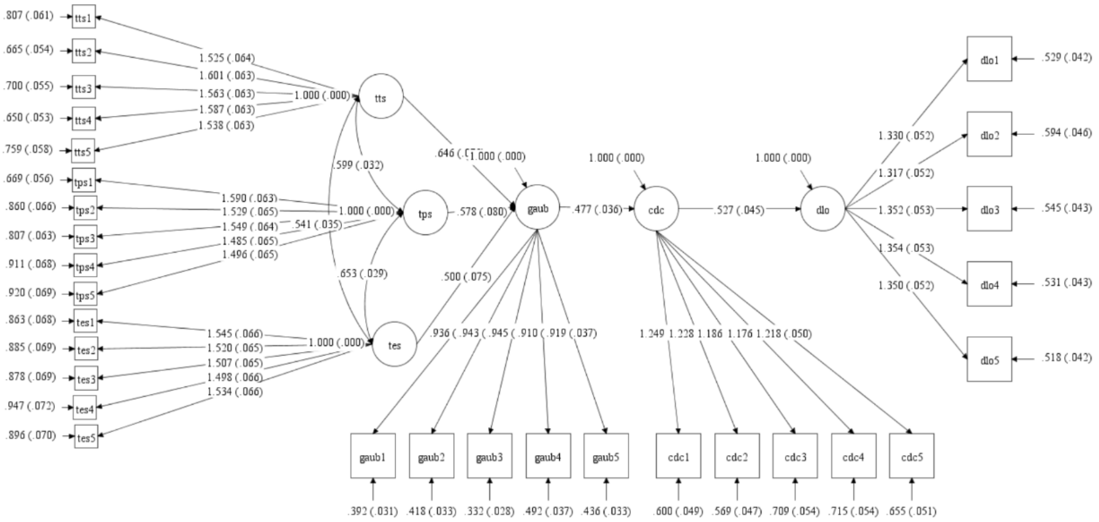

S1 Table. Sample Characteristics and Descriptive Statistics

Variable	Frequency	Percentage (%)	Mean	SD	Skewness	Kurtosis
Gender						
Female	375	75				
Male	125	25				
AI Familiarity						
Very Unfamiliar	50	10				
Unfamiliar	100	20				
Neutral	175	35				
Familiar	125	25				
Very Familiar	50	10				
Construct Scores						
Teacher Technical Support			4.002	1.609	-0.041	-1.093
Teacher Pedagogical Support			4.015	1.585	-0.059	-1.108
Teacher Emotional Support			4.026	1.58	-0.031	-1.104
Generative AI Usage Behavior			4.007	1.685	-0.019	-1.104
Creative Design Capability			3.999	1.632	0.018	-1.065

Design Learning Outcomes			4.026	1.665	-0.087	-1.098

S2 Table. Measurement Model Reliability and Validity

Construct	Items	Factor Loading	t-value	R^2	Cronbach's α	CR	AVE	√AVE
Teacher Technical Support (TTS)					0.932	0.933	0.739	0.86
	TTS1	0.862	65.89	0.742				
	TTS2	0.891	81.82	0.794				
	TTS3	0.882	76.02	0.777				
	TTS4	0.892	82.12	0.795				
	TTS5	0.87	69.88	0.757				
Teacher Pedagogical Support (TPS)					0.925	0.926	0.716	0.846
	TPS1	0.889	78	0.791				
	TPS2	0.855	61.69	0.731				
	TPS3	0.865	65.84	0.748				
	TPS4	0.841	56.65	0.708				
	TPS5	0.842	56.76	0.709				
Teacher Emotional Support (TES)					0.921	0.922	0.702	0.838
	TES1	0.857	61.23	0.734				
	TES2	0.85	58.66	0.723				
	TES3	0.849	58.33	0.721				
	TES4	0.839	54.8	0.703				
	TES5	0.851	59.01	0.724				
Generative AI Usage Behavior (GAUB)					0.963	0.964	0.87	0.933
	GAUB1	0.936	147.53	0.876				
	GAUB2	0.933	141.98	0.871				
	GAUB3	0.946	169.97	0.895				
	GAUB4	0.918	117.57	0.842				
	GAUB5	0.927	131.73	0.86				
Creative Design Capability (CDC)					0.946	0.947	0.785	0.886
	CDC1	0.904	93.79	0.817				

	CDC2	0.906	95.07	0.82				
	CDC3	0.88	76.63	0.774				
	CDC4	0.877	75.11	0.769				
	CDC5	0.892	84.49	0.796				
Design Learning Outcomes (DLO)					0.955	0.956	0.81	0.9
	DLO1	0.912	104.43	0.832				
	DLO2	0.901	94.21	0.812				
	DLO3	0.912	104.86	0.832				
	DLO4	0.914	107.05	0.836				
	DLO5	0.916	108.55	0.839				

S3 Table. Structural Model Results and Hypothesis Testing

Hypothesis	Path	Unstandardized Estimate	SE	t-value	Standardized Estimate	R^2	Support
Direct Effects							
H1a	TTS → GAUB	0.646	0.073	8.852	0.362***		Yes
H1b	TPS → GAUB	0.578	0.08	7.205	0.325***		Yes
H1c	TES → GAUB	0.5	0.075	6.632	0.281***	0.685	Yes
H2	GAUB → CDC	0.477	0.036	13.209	0.648***	0.42	Yes
H3	CDC → DLO	0.527	0.045	11.831	0.569***	0.324	Yes
Correlations							
	TTS ↔ TPS	0.599	0.032	18.932	0.599***		
	TTS ↔ TES	0.541	0.035	15.602	0.541***		
	TPS ↔ TES	0.653	0.029	22.478	0.653***		

Model Fit Indices:χ^2 = 398.373, df = 397, p = 0.471; RMSEA = 0.003 (90% CI: 0.000-0.016); CFI = 1.000; TLI = 1.000; SRMR = 0.023

*Note: **p < 0.001. All hypotheses supported.

S4 Table. Mediation Effects Analysis

Mediation Path	Effect Type	Unstandardized Estimate	SE	t-value	Standardized Estimate	95% CI	Result

H4: Simple Mediation (Teacher Support → GAUB → CDC)							
TTS → GAUB → CDC	Indirect	0.308	0.039	7.982	0.235***	[0.232, 0.384]	Significant
TPS → GAUB → CDC	Indirect	0.276	0.041	6.722	0.210***	[0.196, 0.356]	Significant
TES → GAUB → CDC	Indirect	0.239	0.038	6.249	0.182***	[0.165, 0.313]	Significant
H5: Chain Mediation (Teacher Support → GAUB → CDC → DLO)							
TTS → GAUB → CDC → DLO	Indirect	0.163	0.023	7.021	0.134***	[0.118, 0.208]	Significant
TPS → GAUB → CDC → DLO	Indirect	0.146	0.024	6.115	0.120***	[0.099, 0.193]	Significant
TES → GAUB → CDC → DLO	Indirect	0.126	0.022	5.753	0.103***	[0.083, 0.169]	Significant

Research Ethics Approval

Click here to access/download
Supporting Information
Approval.pdf

Supporting Information-Fig 1

Click here to access/download
Supporting Information
S1_Fig.tiff
