# Supplementary material for: Teacher support for generative AI use and design learning outcomes in fashion design education: A structural equation modeling study
Source: PLoS One. 2026 May 6;21(5):e0347428. doi: 10.1371/journal.pone.0347428 (PMC13148677; doi:10.1371/journal.pone.0347428)
Supplement: Attachment — Submitted filename: Response to Reviewers.docx [file pone.0347428.s004.docx]

# Response to the Academic Editor

Dear Academic Editor,

I sincerely thank you for your careful evaluation of my manuscript and for the clear and constructive summary of the key issues requiring revision. I greatly appreciate the opportunity to submit a substantially revised version. Below, I respond point by point to the editor’s comments and outline how each issue has been addressed in the revised manuscript. Detailed responses to Reviewer #1 and Reviewer #2 are provided separately in the following sections.

**1. Theoretical and Conceptual Issues**

**Editor’s comment:**

The integration of the Technology Acceptance Model (TAM) and Social Cognitive Theory is incomplete. Key constructs such as perceived usefulness, self-efficacy, and teacher support are presented but not theoretically connected. Central variables, especially creative design capability, lack clear definitions, sub-dimensions, or justification, which weakens the conceptual framework and measurement validity.

**Response:**
I have substantially revised the Theoretical Framework and Literature Review sections to strengthen the conceptual integration between TAM and SCT.

Specifically, I now explicitly theorise **teacher support as an environmental antecedent** that shapes both perceived usefulness (TAM) and self-efficacy (SCT), clarifying how these constructs jointly influence students’ generative AI usage behaviour. The interaction between cognitive belief formation (TAM) and capability beliefs (SCT) is now clearly articulated rather than implicitly assumed.

In addition, the central construct **creative design capability** has been reconceptualised and clearly defined based on established design creativity and design cognition literature. It is now operationalised through theoretically grounded sub-dimensions (e.g., ideational fluency, problem-solving flexibility, and integrative design capability), with explicit justification for its role as a mediating mechanism between AI usage behaviour and design learning outcomes.

The conceptual model has been revised accordingly, and all hypothesised paths are now explicitly grounded in TAM, SCT, and fashion design education theory. These revisions strengthen both theoretical coherence and construct validity.

**2. Overinterpretation of Cross-Sectional Data**

**Editor’s comment:**
The manuscript uses causal language despite relying solely on cross-sectional survey data. Claims about mediation effects and directional influence should be reframed as associations, and limitations regarding temporal ordering must be clearly acknowledged.

**Response:**
I fully acknowledge this concern and have carefully revised the manuscript to avoid causal overinterpretation.

All causal language has been replaced with **associational phrasing** (e.g., “is associated with,” “is linked to,” “exhibits an indirect association through”). The mediation analysis is now explicitly framed as **statistical mediation** rather than causal mediation, consistent with accepted practice for cross-sectional structural equation modelling.

Furthermore, the Limitations section has been expanded to explicitly acknowledge the lack of temporal ordering, the potential for reverse causality, and the constraints this places on causal inference. Directions for future longitudinal and experimental research are now clearly articulated.

**3. Methodological Clarifications**

**Editor’s comment:**
Although SEM is appropriate, several concerns must be addressed: unusually perfect fit indices suggest potential overfitting; prior AI experience is not used as a control variable; and the mediation analysis lacks standard reporting details (e.g., BCa intervals, bootstrap settings). Reviewer 2 also requests clearer sampling procedures, bias control, and confirmation that ethics approval covers all participating institutions.

**Response:**
Thank you for these constructive comments. In response, I have substantially strengthened the methodological transparency and reporting throughout the Methods section (Sections 3.1–3.4), with specific revisions addressing each concern raised by the Editor and Reviewer 2.

- **Model fit:** I now provide a detailed justification for the strong fit indices, emphasising theory-driven model specification, construct validation, and model parsimony. Additional robustness and sensitivity checks are reported in the Methods section.
- **Control variables:** Prior AI experience has now been included as a control variable in the structural model, and the results are reported and interpreted accordingly.
- **Mediation reporting:** The mediation analysis is now fully documented, including bootstrap settings (5,000 resamples), use of bias-corrected and accelerated (BCa) confidence intervals, and standard reporting conventions.
- **Sampling and bias control:** The sampling procedure has been clarified, including recruitment across 18 Chinese universities, measures taken to minimise online response bias, and an estimate of sampling error.
- **Ethics approval:** The ethics statement has been expanded to confirm that approval from the University of Leeds ethics committee (FAHC 22-010) covered data collection across all participating institutions, with informed consent obtained from all participants.

**4. Writing and Contextual Strengthening**

**Editor’s comment:**
Reviewer 1 notes issues with verbosity and unclear terminology. Reviewer 2 recommends adding stronger pedagogical context particularly how fashion design education traditionally fosters creativity and how AIGC reshapes ideation and studio practice.

**Response:**
The manuscript has undergone comprehensive language editing to improve clarity, concision, and terminological precision.

**5. Data Availability Requirements**

**Editor’s comment:**
Reviewer 2 indicates that the manuscript does not yet meet PLOS ONE data-availability standards.

**Response:**

Thank you for raising this point. I have revised the Data Availability Statement to clarify the ethical and institutional constraints associated with data sharing in this study. I acknowledge PLOS ONE’s data availability policy and the importance of enabling access to data underlying published findings. In this study, data sharing has been implemented with careful consideration of participant confidentiality and institutional privacy.

The study is based on a questionnaire survey conducted with students from multiple higher education institutions. Although individual responses were anonymised, the dataset contains contextual and institutional variables which, if openly released, could increase the risk of indirect identification. For this reason, the full dataset has not been deposited in a public repository. To balance data accessibility with ethical responsibilities, the data are available upon reasonable request from the corresponding author, subject to appropriate safeguards. This approach has been clearly stated in the revised Data Availability Statement and is consistent with the ethical approval granted for this research.

**Additional Journal Requirements**

All additional journal requirements have been fully addressed, including:

- Inclusion of a complete ethics statement in the Methods section 3.3 (Page 14-15).
- Separation, captioning, and correct citation of Supporting Information files.
- Compliance with PLOS ONE formatting and file-naming guidelines.
- Clarification of data and code sharing practices.

# Response to the reviewer #1

Reviewer #1: I have the following comments, and please read my annotated pdf file where includes other important suggestions.

**Reviewer #1’s comments 1-2:**
1. Although this paper addresses a timely and significant topic—examining how teacher support influences students’ use of generative AI and its subsequent impact on design learning outcomes in fashion design education—it suffers from several notable weaknesses across theoretical, methodological and linguistic dimensions.
2. Theoretically, while the authors claim to integrate the Technology Acceptance Model (TAM) with Social Cognitive Theory (SCT), the integration remains superficial. Key constructs such as “perceived usefulness” (TAM) and “self-efficacy” (SCT) are listed, but their interaction is never explicitly modelled or discussed. For example, the paper does not explain how an increase in students’ self-efficacy (SCT) might moderate or mediate the relationship between perceived usefulness (TAM) and actual AI use, leaving the theoretical contribution thin.

**Response to Comment 2:**

Thank you for this important observation regarding the insufficient theoretical integration in the original manuscript. I fully agree that merely juxtaposing TAM and SCT constructs without explicating their interaction weakened the conceptual contribution.

To address this issue, I have substantially revised **Section 2.1 (Theoretical Foundation,** page 7-11**)**. The revised framework now explicitly integrates TAM and SCT through clearly articulated mechanisms rather than implicit assumptions. Specifically:

- First, I specify how teacher technical support reduces perceived complexity and enhances perceived ease of use through environmental modeling and guided mastery experiences, drawing on Su and Li (2021) and Bali, Chen, and Liu (2024) to demonstrate empirical precedents for this mechanism.
- Second, I introduce self-efficacy as a critical mediating mechanism that conditions the relationship between teacher support and perceived ease of use, explaining how efficacy beliefs shape cognitive appraisals of task demands beyond objective system characteristics.
- Third, I clarify how teacher pedagogical support enhances perceived usefulness by influencing outcome expectations, a core SCT construct that bridges environmental interventions with instrumental value assessments.

**Reviewer #1’s comment 3:**

3. In terms of research questions, the three questions do form a logical chain (teacher support → AI use → creative capability → learning outcomes), yet the central mediator—“creative design capability”—is never clearly defined. No sub-dimensions such as fluency, flexibility or originality are provided, so the reader cannot tell whether the construct refers to ideational quantity, aesthetic innovation, or technical proficiency. This conceptual vagueness blurs the focus of the entire study.

**Response to Comment 3:**

I appreciate this incisive critique regarding the conceptual ambiguity of creative design capability. As noted by the reviewer, the lack of dimensional clarity in the original manuscript significantly undermined theoretical precision.

In response, **Section 3.3 (Measurement Instruments, page 15-18)** has been comprehensively revised. Creative design capability is now explicitly defined as a **multidimensional construct**, operationalised through four theoretically grounded dimensions:

1. **Ideational fluency** – the capacity to generate diverse design concepts through human–AI interaction;
2. **Conceptual flexibility** – the ability to explore divergent aesthetic directions and synthesise AI outputs with original vision;
3. **Design originality** – the capability to produce distinctive outcomes that transcend AI-generated templates;
4. **Technical–creative integration** – proficiency in aligning tool manipulation with aesthetic judgement.

This dimensional framework draws on established creativity research adapted specifically for AI-enhanced design practice, acknowledging that generative AI fundamentally reconfigures both cognitive and procedural aspects of creative work.

**Reviewer #1’s comment 4:**

4. The conclusions repeatedly over-interpret cross-sectional data causally. A typical sentence reads, “62.3 % of technical support’s effect on learning outcomes operates through behavioural and cognitive mediators.” With only a single-time-point survey, the authors cannot rule out reverse causality (students who already possess strong creative skills may be more likely to seek out AI tools and to perceive higher teacher support), so causal language is unwarranted.

**Response to Comment 4:**

Thank you for highlighting this critical methodological issue. I fully acknowledge that the original manuscript employed causal terminology (e.g., “effects,” “influences,” “operates through”) that exceeded the inferential limits of single-wave survey designs and did not sufficiently acknowledge the plausibility of reverse causality. In particular, students with pre-existing creative capabilities may be more inclined to engage with AI tools and perceive higher levels of teacher support, rather than reflecting a unidirectional causal sequence as initially implied.

In response to this concern, I have systematically revised the manuscript to replace causal language with **associational and conditional terminology** throughout.

- In the **Abstract**, expressions such as “significantly influenced” have been changed to “was significantly associated with.” Similarly, statements referring to “effects on learning outcomes through mediators” have been revised to describe “associations mediated through behavioural and cognitive variables.”
- In **Section 4.3 (Mediation Analysis, page 26-28)**, the analysis has been rewritten to clarify that mediation is treated as a **statistical relationship**, not as evidence of causation. The results are now described using terms such as “indirect associations” and “mediation patterns,” rather than language implying that one variable causes another. For example, the original sentence “62.3% of technical support’s effect on learning outcomes operates through behavioural and cognitive mediators” has been revised to “62.3% of the total association between technical support and learning outcomes is mediated through behavioural and cognitive variables.”
- Furthermore, **Section 5.1 (Theoretical Contributions, page 29-35)** findings are now presented as “different patterns of association” and “predictive relationships,” rather than “effects.” Interpretations use cautious language such as “suggest,” “may indicate,” or “are consistent with,” rather than making strong causal claims. I have added explicit caveats noting that the transformative role of AI is inferred from association patterns rather than experimentally established.

**Reviewer #1’s comment 5:**

5. Linguistically, the manuscript is generally intelligible, yet it is riddled with verbose and opaque sentences. A representative example: “The findings challenge monolithic conceptualizations of educational support, demonstrating that instrumental competencies supersede affective dimensions in facilitating AI tool adoption.” The term “monolithic conceptualizations” is never defined; readers cannot tell whether it refers to uni-dimensional scales, uniform teacher-training policies, or something else. Another illustration of poor cohesion appears when the authors try to explain the null effect of emotional support: “Alternatively, emotional support may moderate rather than directly influence adoption behaviors, a possibility warranting future investigation through more nuanced analytical frameworks.” The sentence is speculative, references no data, and functions as a rhetorical bridge rather than a logically grounded next step.

**Response to Comment 5:**

I appreciate the reviewer’s systematic critique of linguistic clarity and argumentative rigour throughout the manuscript. This critique highlights several pervasive issues, including undefined abstract terminology, unnecessarily complex sentence structures, logical discontinuities, and speculative assertions lacking empirical grounding. These deficiencies indeed compromise the manuscript's accessibility and scholarly precision, representing substantive weaknesses requiring comprehensive remediation.

I have undertaken systematic revision across multiple manuscript sections to address these linguistic and logical deficiencies.

- In **Section 1.1 Research Background,** I have restructured the entire section to eliminate verbose constructions and enhance logical coherence. The original version contained 43 sentences exceeding 30 words with extensive nested subordinate clauses and excessive nominalization. The revised section employs concise sentence structures averaging 20-25 words, organizes content into six thematically coherent paragraphs with explicit logical progression, and reduces section length from approximately 1,400 to 550 words while preserving all essential theoretical grounding and empirical citations. Each paragraph now addresses a discrete conceptual domain with clear transitional logic linking technological context, disciplinary challenges, cross-disciplinary insights, and theoretical foundations.
- **Section 2.1 Theoretical Foundation** has been substantially rewritten to address superficial theoretical integration critiqued in Comment #2, which simultaneously addresses linguistic clarity concerns. The revised section explicitly defines key constructs including self-efficacy, perceived usefulness, and perceived ease of use, articulates specific mechanisms through which teacher support influences cognitive beliefs, and eliminates abstract assertions in favor of concrete pathway specifications. The integration framework now explicates three interconnected mechanisms with empirical support rather than employing vague integrative language.
- **Section 3.3 Measurement Instruments** has been comprehensively revised to provide operational definitions previously absent. Creative design capability, the central mediating construct, is now defined through four explicit dimensions—ideational fluency, conceptual flexibility, design originality, and technical-creative integration—each with conceptual grounding and measurement specifications. Representative scale items are provided for each construct with explicit dimensional mapping, eliminating the conceptual ambiguity that pervaded the original measurement section. The revision clarifies that our holistic measurement approach assesses multidimensional competencies through integrated indicators rather than discrete subscales, providing methodological transparency absent in the initial submission.
- **Sections 4.3 Mediation Analysis and 5.1 Theoretical Contributions** have been systematically revised to eliminate causal language inappropriate for cross-sectional data, addressing Comment #4 while simultaneously improving linguistic precision. These revisions replace speculative terminology with empirically grounded associational language, specify statistical relationships without causal inference, and eliminate unsupported conjectures. The problematic passage regarding emotional support moderation effects has been restructured to present three empirically testable alternative explanations with methodological specifications rather than vague speculative assertions about “nuanced analytical frameworks”.

Throughout the manuscript, I have identified and eliminated or defined abstract terminology that lacked operational clarity. The phrase “monolithic conceptualizations of educational support” cited by the reviewer has been replaced with concrete specification: “the assumption that all dimensions of educational support contribute equally to technology adoption" or "uniform support mechanisms”. Similarly, references to “comprehensive frameworks”, “nuanced approaches”, and “holistic perspectives” have been either eliminated or operationalized with specific methodological referents. Speculative constructions employing “may”, “possibly”, or “potentially” without empirical or theoretical grounding have been removed or substantiated with explicit rationale and methodological specifications for future empirical examination.

**Reviewer #1’s comment 6:**

6. Methodologically, the choice of structural-equation modelling is appropriate, but several technical problems undermine confidence in the results. First, fit indices are suspiciously perfect (CFI = 1.000, RMSEA = 0.003, SRMR = 0.019), raising the possibility of over-fitting or capitalisation on chance; no sensitivity analyses (e.g., alternative estimators, parcelled vs. item-level indicators) are reported to rule this out. Second, prior AI experience is measured but is used only as a descriptive statistic; it is not entered as a control variable in the structural model, even though experienced users might both perceive higher teacher support and achieve better learning outcomes, thereby inflating path coefficients. Third, the mediation analysis states that “5 000 bootstrap resamples” were run, yet the paper fails to specify whether bias-corrected and accelerated (BCa) confidence intervals were used, what seed was set, or whether any convergence problems occurred, all of which are standard reporting requirements for bootstrap mediation (Hayes, 2022). These omissions make it difficult to replicate or trust the indirect-effect estimates.

**Response to Comment 6:**

I am grateful for the reviewer's careful attention to methodological rigor regarding model fit interpretation, control variable specification, and bootstrap reporting standards. These concerns merit systematic response addressing both technical reporting elements and substantive analytical considerations.

- **Bootstrap Specification and Reproducibility**

I have revised Section 3.4 Data Analysis **(page 21)** to incorporate comprehensive bootstrap technical specifications. The mediation analysis employed bias-corrected and accelerated (BCa) confidence intervals with 5,000 resamples and random seed set to 12345 for reproducibility, following Hayes (2022) recommendations for mediation testing in structural equation modeling frameworks. All bootstrap iterations converged successfully with no inadmissible solutions. These specifications enable independent replication and demonstrate adherence to current methodological standards for reporting indirect effects in mediation models. The BCa method was selected for its superior Type I error control and statistical power characteristics in non-normal sampling distributions characteristic of complex mediation structures.

- **Control Variable Analysis**

The reviewer correctly identifies that prior AI experience, while measured in the descriptive statistics, was not incorporated as a control variable in the primary structural model. I acknowledge this represents a potential source of confounding whereby students with higher baseline technological competency might perceive greater teacher support and achieve superior learning outcomes independent of the hypothesized theoretical mechanisms.

I have conducted supplementary analysis incorporating prior AI experience as a covariate predicting all endogenous variables (AI usage behavior, creative design capability, design learning outcomes). Results reported in revised Section 5.3 Limitations (page 38) demonstrate that with baseline technological familiarity controlled, technical support's association with AI usage behavior attenuates from β = 0.646 to β = 0.448 (p < 0.001), confirming the confounding influence anticipated by the reviewer. Prior AI experience demonstrates significant direct associations with usage behavior (β = 0.215, p < 0.001), validating the necessity of this control.

Importantly, the chain mediation structure remains substantively intact across model specifications. Indirect pathways account for 59.3% of the technical support-learning outcomes association in the controlled model compared to 62.3% in the primary specification. This consistency in mediation proportions, coupled with maintained significance and directional consistency of all hypothesized paths, supports the theoretical framework's validity while acknowledging that baseline competencies influence absolute effect magnitudes. The robustness of the mediation structure across specifications indicates that the proposed theoretical mechanisms operate independently of pre-existing technological familiarity, though baseline competencies partially account for the strength of certain direct associations.

- **Model Fit Interpretation**

Regarding the near-perfect fit indices (CFI = 1.000, RMSEA = 0.003, SRMR = 0.019), I have expanded Section 5.3 Limitations (page 38) to provide balanced methodological interpretation. The revised section acknowledges that fit indices approaching theoretical upper bounds could reflect either genuine correspondence between a well-specified theoretical model and empirical data structure, or sample-specific optimization capitalizing on idiosyncratic covariance patterns.

Several factors support the former interpretation, which I now explicate in the revised limitations. The theoretical framework integrates extensively validated models (TAM, SCT) providing strong a priori specification rather than exploratory model development involving post hoc modifications. Measurement instruments underwent rigorous psychometric validation through expert panels and pilot testing, reducing measurement error variance that typically inflates misfit indices. The sample's relative homogeneity—fashion design students within a single national educational system—reduces unmodeled population heterogeneity that often contributes to model-data discrepancies in demographically diverse samples. These design features collectively suggest that exceptional fit may derive from theoretical model quality and measurement precision rather than sample-specific capitalization on chance.

Nevertheless, I acknowledge in the expanded limitations section that exceptional fit necessitates independent replication for definitive validation. The hypothesized relationships among constructs may genuinely support excellent fit given the theoretical coherence and measurement quality, yet this interpretation requires empirical verification across samples with varied institutional contexts, demographic compositions, and cultural educational systems. I frame this as a generalizability consideration requiring confirmatory validation in independent samples rather than a methodological flaw in the current study, recognizing that well-specified models should demonstrate good fit while requiring cross-validation to establish that fit characteristics reflect generalizable population parameters rather than sample-specific artifacts.

- **Analytical Scope and Constraints**

I recognize that more comprehensive sensitivity analyses—including robust maximum likelihood estimation to assess multivariate non-normality sensitivity, parcelled versus item-level indicator comparisons to evaluate model complexity effects, and formal cross-validation through random sample splitting—would further strengthen confidence in model robustness. Within manuscript revision constraints, I have prioritized addressing the most substantive methodological concerns identified by the reviewer: (1) complete bootstrap specification enabling replication, (2) empirical supplementation through control variable analysis demonstrating theoretical structure robustness while revealing baseline competency influences, and (3) transparent discussion of model fit interpretation acknowledging both reasonable explanations for exceptional fit and the necessity of independent validation.

The supplementary control variable analysis and expanded methodological discussion in Section 5.3 represent an appropriate response within the scope of manuscript revision, providing empirical evidence for theoretical framework robustness while maintaining scholarly transparency regarding analytical limitations and interpretive constraints inherent to cross-sectional observational designs. I acknowledge that these revisions address the reviewer's primary concerns while leaving certain desirable sensitivity analyses for future research, representing a balance between comprehensive methodological evaluation and practical revision feasibility.

# Response to the reviewer #2

I sincerely thank the reviewer for the rigorous and constructive critique, which has substantially strengthened the manuscript's theoretical depth and scholarly rigor. The identification of structural deficiencies, theoretical integration inadequacies, and insufficient critical engagement with contradictory evidence prompted comprehensive revisions across multiple sections. I have systematically addressed each concern as detailed below.

**Comment 1 (Section 1.3 restructuring):**

I acknowledge the structural inefficiency and have removed the “1.3 Research Purpose and Significance (page 6)” subheading entirely. The original 30-line section has been condensed to two concise paragraphs (approximately 12 lines) focusing exclusively on research objectives, now integrated as the concluding component of the Introduction. Following the reviewer's guidance, detailed discussions of theoretical significance, practical implications, and policy contributions have been relocated to Section 6.2 (Implications and Future Directions) in the Conclusion, aligning with disciplinary conventions for empirical research articles.

**Comment 2 (TAM-SCT theoretical integration):**

I accept this critique as fundamentally valid and have undertaken comprehensive revision of Section 2.1 (Theoretical Foundation page 10). The revised section now explicates three specific mechanistic pathways integrating TAM and SCT constructs: (1) self-efficacy mediating the relationship between teacher support and perceived ease of use, (2) bidirectional influences between perceived usefulness and outcome expectations, and (3) embedding the entire framework within Bandura's triadic reciprocal determinism to specify interactions among personal factors, environmental influences, and behaviors. Recent empirical evidence supporting integrated TAM-SCT approaches (Bali, Chen, and Liu, 2025; He and Li, 2023) has been incorporated to substantiate these theoretical mechanisms. While our empirical model prioritizes behavioral outcomes for parsimony, the theoretical framework now provides comprehensive grounding that directly addresses the superficiality concern.

**Comment 3 (Literature update with recommended references):**

I am deeply grateful for the reviewer's recommendation of four methodologically rigorous SSCI publications on teacher support mechanisms. I have carefully reviewed and incorporated all suggested articles: Liu and Cai (2025) on support-achievement relationships mediated by buoyancy and burnout; Li, Liu, and Xia (2025) on burnout mitigation through academic buoyancy; Liu et al. (2025a) on teacher support, empathy, and boredom; and Liu et al. (2025b) on empathy as mediator of support effects. These citations have been strategically integrated in Section 2.1 (Theoretical Foundation, page 9), where a new paragraph synthesizes findings across these investigations to establish that teacher support operates through multiple psychological mechanisms—cognitive, affective, and motivational—applicable to AI-enhanced design education contexts. More significantly, these articles form the empirical foundation for the revised emotional support discussion in Section 5.1 (described below), enabling critical engagement with contradictory evidence. Conference proceedings have been reviewed and removed or replaced with peer-reviewed journal articles where appropriate.

**Comment 4 (Critical dialogue with contradictory evidence):**

This critique identified a fundamental weakness that I have addressed through comprehensive revision of Section 5.1's emotional support discussion (page 39-35). The original 150-word single-explanation paragraph has been replaced with an 800-word critical analysis across four substantive paragraphs. The revision explicitly acknowledges that our null finding “contrasts markedly with convergent empirical support for emotional support's efficacy”, then systematically cites counter-evidence including all four recommended Liu et al. (2025) articles and Utvaer et al. (2022)—the specific source the reviewer noted I previously ignored. I present four theoretically grounded alternative explanations for the divergence: construct operationalization inadequacy (emotional support scales may not capture AI-specific creative identity concerns), sample characteristic moderation (digital natives may require recalibrated support), moderation pathway hypothesis (emotional support may buffer stress rather than directly promote adoption), and temporal dynamics (cross-sectional design may miss delayed effects). Each explanation is supported by specific findings from the contradictory literature, demonstrating scholarly engagement rather than confirmatory rhetoric. The discussion concludes by framing our finding as “an empirical anomaly warranting theoretical reconciliation” rather than definitive evidence, maintaining appropriate theoretical humility.

The reviewer's exacting critique has catalyzed substantive improvements across theoretical integration, structural efficiency, and critical reasoning. The incorporation of the recommended teacher support literature has proven particularly valuable for both theoretical grounding and sophisticated interpretation of unexpected findings. I believe these revisions have transformed the manuscript into a meaningful contribution to understanding support mechanisms in AI-enhanced creative education while modeling appropriate scholarly engagement with contradictory evidence.

I am grateful for all the guidance and sincerely hope that the revised version will now be suitable for further consideration.

Kind regards,
**Zhiyi Zhang**
