# Supplementary material for: Teacher support for generative AI use and design learning outcomes in fashion design education: A structural equation modeling study
Source: PLoS One. 2026 May 6;21(5):e0347428. doi: 10.1371/journal.pone.0347428 (PMC13148677; doi:10.1371/journal.pone.0347428)
Supplement: Attachment — Submitted filename: Response to Editor and Reviewers.docx [file pone.0347428.s005.docx]

Dear Dr. Carels and Editorial Team,

Thank you for your continued support of our manuscript entitled "Teacher Support for Generative AI Use and Design Learning Outcomes in Fashion Design Education: A Structural Equation Modeling Study" (PONE-D-25-50100R1), and for coordinating the review process so efficiently.

I am grateful to both reviewers for their constructive engagement with our work. I am pleased that Reviewer #1 and Reviewer #2 found the revised manuscript satisfactory and recommended it for publication. Their detailed and thoughtful feedback in the previous round substantially strengthened the quality of this work, and I sincerely appreciate their time and expertise.

In response to the two outstanding requirements noted in your decision letter, I have made the following updates:

1. Retracted Reference: The retracted article (Xu & Jiang, 2022; https://dl.acm.org/doi/10.1145/3526219) has been removed from the reference list, and all in-text citations to this work have been deleted accordingly. The reference list has been renumbered to reflect this change.

2. IRB Documentation: The data reported in this manuscript derive from a research component undertaken within my broader doctoral project on fashion design education, for which ethical approval had already been granted. Accordingly, the approved study protocol and related ethics documentation from that doctoral project, under which the present study was covered, have been uploaded as an “Other” file for editorial review in accordance with PLOS ONE’s requirements for human subjects research ethics.

A revised manuscript with tracked changes, a clean final version, and this response letter are submitted herewith.

I hope that the revised submission now fully meets PLOS ONE's publication criteria, and I look forward to hearing from you.

Yours sincerely,

Zhiyi Zhang
